# Reconstruction of Tissue-Specific Metabolic Networks Using CORDA

**DOI:** 10.1371/journal.pcbi.1004808

**Published:** 2016-03-04

**Authors:** André Schultz, Amina A. Qutub

**Affiliations:** Department of Bioengineering, Rice University, Houston, Texas, United States of America; The Pennsylvania State University, UNITED STATES

## Abstract

Human metabolism involves thousands of reactions and metabolites. To interpret this complexity, computational modeling becomes an essential experimental tool. One of the most popular techniques to study human metabolism as a whole is genome scale modeling. A key challenge to applying genome scale modeling is identifying critical metabolic reactions across diverse human tissues. Here we introduce a novel algorithm called Cost Optimization Reaction Dependency Assessment (CORDA) to build genome scale models in a tissue-specific manner. CORDA performs more efficiently computationally, shows better agreement to experimental data, and displays better model functionality and capacity when compared to previous algorithms. CORDA also returns reaction associations that can greatly assist in any manual curation to be performed following the automated reconstruction process. Using CORDA, we developed a library of 76 healthy and 20 cancer tissue-specific reconstructions. These reconstructions identified which metabolic pathways are shared across diverse human tissues. Moreover, we identified changes in reactions and pathways that are differentially included and present different capacity profiles in cancer compared to healthy tissues, including up-regulation of folate metabolism, the down-regulation of thiamine metabolism, and tight regulation of oxidative phosphorylation.

## Author Summary

Cellular metabolism is defined by a large, intricate network of thousands of components, and plays a fundamental role in many diseases. To study this network in its entirety, metabolic models have been built which encompass all known biochemical reactions in the human metabolism. However, since not all metabolic reactions take place in any given tissue, these generalized models need to be tailored to study specific cell types. Algorithms developed to date to perform this tailoring process have focused on keeping tissue-specific models as concise as possible. This approach, however, can remove essential reactions from the model and hamper subsequent analysis. Here we present CORDA, a tissue-specific building algorithm that yields concise, but not minimalistic, tissue-specific models. CORDA has many advantages over previous methods, including better agreement with experimental data and better model functionality. Using CORDA, we developed a library of 76 healthy and 20 cancer-specific models of metabolism, which we used to identify similarities between healthy and cancerous tissues, as well as metabolic pathways that are unique to cancer. Results of this work provide a broadly applicable tool to model cell- and tissue-specific metabolism, while highlighting potential new pathway targets for cancer therapies.

## Introduction

Genome-wide Metabolic Reconstructions (GEMs) computationally model the molecules and reactions responsible for metabolism in any given organism, and have been applied across a variety of fields including metabolic engineering and evolutionary analysis [1]. Computational methods developed to study GEMs [2] have generated novel hypotheses about the structure of metabolic networks in microorganisms, and helped elucidate gaps in our knowledge of metabolism [3, 4]. Since the publication of the comprehensive human metabolic reconstruction Recon1 [5], human GEMs have enabled the study of human metabolism at a genome level [6]. These studies include the prediction of novel metabolic functions [7], prediction of metabolic biomarkers for congenital genetic disorders [8, 9], context analysis of omics data [10–12], comparison between humans and other mammals through gene homolog mapping [13, 14], and prediction of suitable cancer drugs [15, 16] and drug targets [17–19].

A particularly prolific subfield of human GEMs is the development of tissue-specific reconstructions. Different groups of metabolic reactions occur in different cell types. Hence, numerous studies have been dedicated to generating tissue specific or cell specific models of metabolism [20, 21]. These tissue-specific reconstructions can be built by piecing together the model based on previously established biological evidence obtained by reviewing the literature [22–26], through the integration of omics data and computational methods in order to tailor generic, published human reconstructions [5, 9, 27–29] to the desired cell type [15, 16, 30–33], or through a combination of computational algorithms and manual curation [27, 28, 34–36].

Automated tissue-specific reconstruction algorithms developed to date can be broadly categorized into two groups [20]: “flux-dependent” and “pruning” methods. Flux dependent methods find an optimal flux distribution through the general reconstruction which contains the maximum number of high confidence reactions (i.e. reactions whose presence is supported by significant experimental data) [15, 31, 32, 37–39]. These algorithms have been successfully used to predict gene essentiality in cancer tissues [19, 33], cancer specific metabolic pathways [31], metabolic biomarkers for congenital genetic disorders [8, 9], and cancer specific anti-growth factors [15, 16]. One of the main advantages of flux-dependent methods is the fact that they predict a flux distribution along with the tissue-specific model [20]. While this characteristic can be desirable, it also renders flux-dependent reconstructions “snapshots” of the metabolic state defined by the data, as opposed to comprehensive, functional metabolic models [15, 20].

The second category of tissue-specific reconstruction methods are pruning algorithms, which include MBA [34], mCADRE [30] and fastCORE [40]. Models generated using these algorithms have been used to calculate metabolic flux values in hepatocytes [34], identify pathways specific to cancer [30], and predict cancer drug targets [17, 18]. These algorithms start with a core set of reactions, obtained through literature review or experimental data, and proceed by removing the remaining reactions in the generalized human reconstruction while maintaining functionality in the core set. In these algorithms, a tradeoff can be defined between maintaining the model as concise as possible and including all core reactions. That is, if a core reaction requires too many undesirable reactions to carry flux, the algorithm may remove this core reaction from the tissue model, a tradeoff referred to as *flexible core*.

There are two main advantages to defining a core set of reactions before performing the tissue-specific algorithm. The first advantage is the possible inclusion of multiple sources of data and biochemical information [20, 34]. The definition of the reactions core is left to the user’s discretion, allowing for both the combination of data sources and the manual inclusion of reactions. Secondly, reactions with overwhelming evidence are always included in the final tissue model, since a non-flexible set of high confidence reactions can be defined [20]. This pruning approach then allows for the construction of comprehensive tissue models, containing all reactions that may be in a tissue’s metabolism, as opposed to a snapshot of the metabolic state returned by the flux-dependent methods [15, 20].

**Fig 1:**
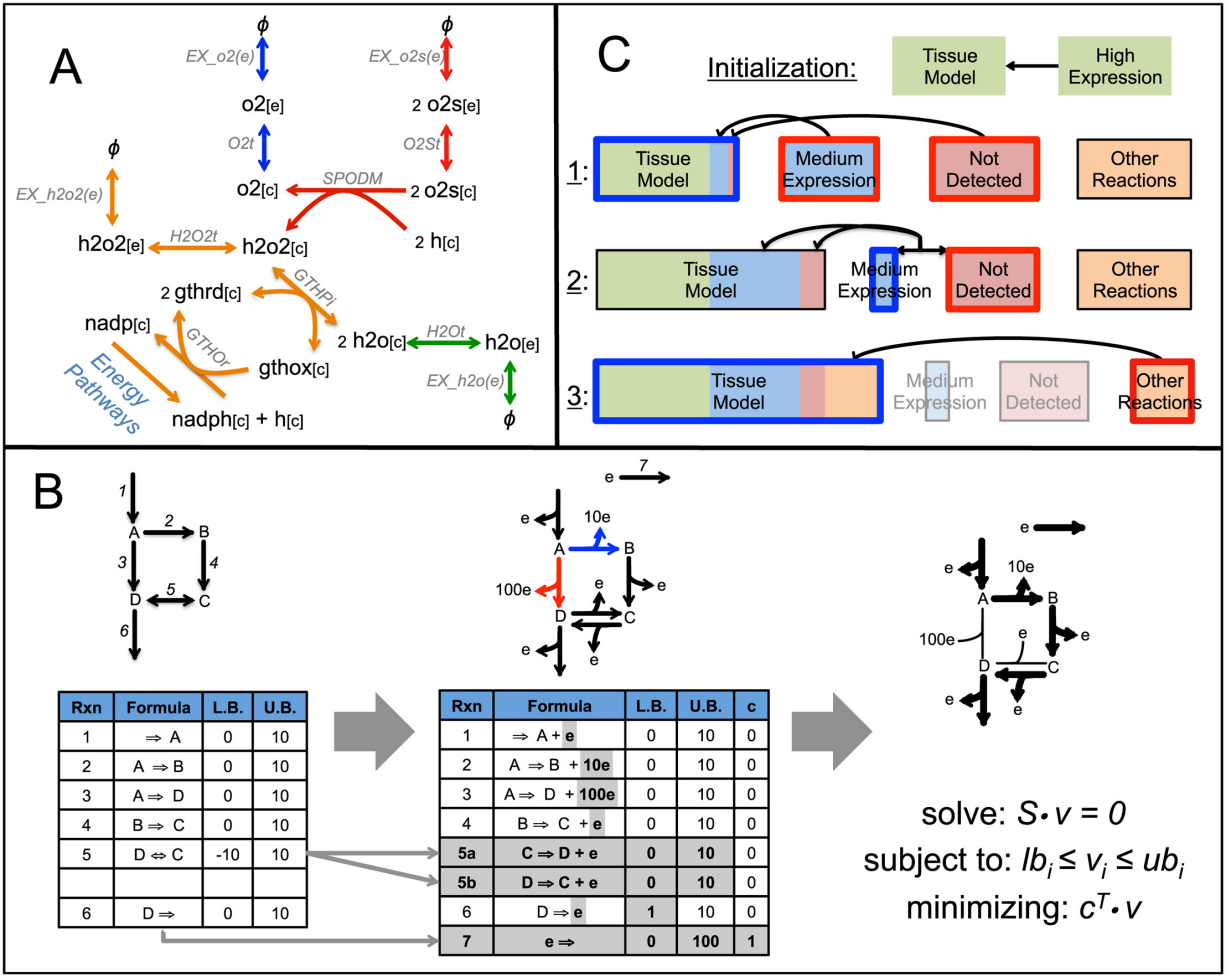
Overview of the CORDA algorithm. (A) Recon1 subnetwork involving water (h2o), oxygen (o2), hydrogen peroxide (h2o2) and superoxide anion (o2s) illustrating how standard oxygen (blue) and water (green) import pathways can be substituted by alternative, physiologically unlikely pathways (red and orange respectively). All metabolites and reactions are labeled as in Recon1. (B) Overview of the *dependency assessment* method. Each reaction in the reconstruction is associated with a specific cost through the addition of a pseudo-metabolite to the model. FBA is then performed while minimizing the cost production in order to identify high cost reactions which are favorable to the reaction being tested. (C) The CORDA tissue-specific algorithm. During each step, reaction groups being tested are outlined in blue, while reaction groups associated with a high cost are outlined in red.

Current pruning methods are also accompanied by two major limitations, however. First, the order in which reactions are removed from the model plays a major role in the final reconstruction. Second, similar to flux-dependent methods, current algorithms aim to keep the final tissue-reconstruction as concise as possible, an approach referred to as *parsimonious*. These algorithms aim to remove from the tissue-specific model all reactions for which experimental data is unsupportive or unavailable, such as reactions with low levels of gene expression or non-gene associated reactions. While a concise tissue-specific reconstruction is desirable, keeping the reconstruction as parsimonious as possible may lead to the removal of fundamental reactions and physiologically unlikely flux distributions. In Recon 1, for instance, oxygen and H_2_O exchange reactions can be removed from the reconstruction with no effect on model functionality (Fig 1A). During simulations, however, these would be replaced by the uptake of the toxic metabolites superoxide anion and hydrogen peroxide respectively, leading to the prediction of physiologically inaccurate flux distributions (Fig 1A). The oxygen exchange reaction is in fact not present in the MBA and mCADRE liver reconstructions, and the water exchange reaction is not present in the mCADRE liver reconstruction.

Hence, in order to ensure our algorithm did not rely on alternative, physiologically unlikely pathways, and that it was independent of any ordering assignments, we chose to take an approach which was not parsimonious. Here we introduce a novel tissue-specific reconstruction algorithm based on Cost Optimization Reaction Dependency Assessment (CORDA). CORDA returns a concise, functional tissue-specific reconstruction, and features a flexible reactions core. CORDA does not depend on Flux Variability Analysis [41] or Mixed Integer Linear Programming (MILP) problems, but only on Flux Balance Analysis [42] (FBA), which is dependent on Linear Programming (LP). This characteristic renders CORDA considerably faster than previous, similar methods. Finally, the CORDA algorithm returns reaction associations that assist in any manual curation to be performed following the automated reconstruction process.

In line with previous studies [43], we apply CORDA to generate a library of 76 healthy and 20 cancer-specific metabolic reconstructions. These reconstructions enabled us to identify metabolic similarities amongst healthy tissues as well as key differences between healthy and cancerous tissues. Furthermore, by sampling the feasible solution space in cancer and healthy models, this library can be used to predict the up- and down-regulation of cancer-specific pathways in cancer metabolism.

## Results

### The CORDA algorithm

The CORDA algorithm is based on a novel approach to identify the dependency of desirable reactions (i.e. reactions with high experimental evidence) on undesirable reactions (i.e. reactions with no experimental evidence), a method referred to here as *dependency assessment*. In the *dependency assessment* approach, the metabolic network is modified in four ways (Fig 1B). First, reversible reactions are split into forward and backward components. Second, a pseudo-metabolite is added as a product for every reaction in the model. At this point, undesirable reactions will carry a higher stoichiometric coefficient for this added metabolite, assigning these reactions a higher “cost”. Third, a reaction consuming this pseudo-metabolite is added to the model. Finally, a positive lower bound is set for the reaction being tested in order to force that reaction to carry flux. After modifying the network, FBA (Materials and Methods) is performed while minimizing the flux through the cost-consuming reaction (Fig 1B). The flux distribution returned will then use high cost, undesirable reactions only as necessary for the reaction being tested to carry flux. Throughout the manuscript, we will refer to high cost reactions predicted to carry flux as *associated* with the reaction being tested. In order to identify pathways with the same cost (i.e. same number of undesirable reactions), multiple dependency assessment can be performed while adding a small amount of noise to the cost of each reaction.

Using this *dependency assessment*, we have developed the CORDA algorithm for the reconstruction of tissue-specific models (Fig 1C). CORDA takes as input the reactions in the generalized human reconstruction separated into high (HC), medium (MC), and negative (NC) confidence groups (see Materials and Methods section for a detailed description). All remaining reactions in the reconstruction (i.e. non gene associated reactions or reactions for which no data is available) are designated as others (OT). All HC reactions are included in the model, and the maximum number of MC reactions is included while minimizing the inclusion of NC reactions. While the definition of these four reaction groups are left to the user’s discretion, here we categorize them according to proteomics data from the Human Protein Atlas (HPA)[44, 45] and a methodology used in previous studies [30, 32, 37] (Materials and Methods). To begin the algorithm, all HC reactions are moved into the tissue reconstruction (RE). In a first step, MC and NC reactions associated with each RE reaction (which are the same as the HC group at this point) are identified using the dependency assessment and moved into the RE group. In a second step, NC reactions associated with a high number of MC reactions are identified and moved into the tissue model, and all remaining NC reactions are blocked (upper and lower bounds set to zero). Next, all MC reactions still able to carry flux are also moved to the RE group. Finally, in the final step of the algorithm, all OT reactions associated with any RE reaction are moved to the RE group for the final tissue-specific model. A detailed description of the CORDA method, including detailed steps, algorithm parameters, and categorization of model reactions is available in the Materials and Methods section.

### Validation of the CORDA algorithm

#### Parameter sensitivity analysis

As a first step in the validation of the CORDA algorithm, we generated 108 hepatocyte specific models using a wide range of algorithm parameters (Materials and Methods). The data used in this step, as well as the generalized human reconstruction, were the same used during the mCADRE liver reconstruction to allow for a fair and direct comparison between models. The 108 calculated models have an average of 1,857.3 (±21.0) reactions, 1,760 (94.8%) of which are present in all models. Also, 98.3% of all MC reactions are present in all models, and 96.2% of the flexible MC and NC reactions core is unanimously determined as either present or not present in all 108 models. A small number of NC reactions (20.25%) was also present in all reconstructions. Interestingly, the protein or expression evidence for half of those NC reactions has changed in the HPA since the publication of the mCADRE model, and they are no longer considered not detected. This demonstrates the ability of CORDA to include essential, significant NC reactions in the tissue-specific model, as well as the importance of a flexible core.

The main difference between the 108 calculated models stems from the number of OT reactions included. Reconstructions calculated using multiple dependency assessments, in order to identify pathways with the same cost, led to the inclusion of more OT reactions during the final step, defining a tradeoff between model size and robustness. No other parameter generated significantly different reconstructions, and all reconstructions demonstrated significant robustness to parameter values. More information on this analysis is available in the supplemental information (S1 Text).

#### Cross-validation

In order to assess CORDA’s ability to include relevant reactions in the tissue-specific model, we performed an additional 100 cross validation reconstructions using randomly sampled subsets of each reaction group. For each reconstruction performed in this analysis, a subset of 80% of each reaction group used to calculate the previous 108 reconstructions was sampled and used in the same reconstruction process. For each model generated, a hypergeometric p-value was calculated for each reaction group based on how many of the reactions randomly left out during the reconstruction process were ultimately included in the tissue model. The 100 p-values obtained were then combined using Fisher’s method, showing that the tissue-specific models generated here were enriched in a statistically significant manner with the HC and MC reactions left out of the reconstruction process, but not with the NC reactions. This analysis demonstrates the ability of CORDA to selectively include reactions with supportive experimental data. Further information on the cross-validation analysis is available in S1 Text.

#### Comparison to previous models

As further validation, the CORDA algorithm was compared to two previously published methods: MBA [34] and mCADRE [30]. MBA and mCADRE were selected for comparison because they both contain a flexible core feature, and are both pruning algorithms, returning a comprehensive tissue-specific reconstruction like CORDA. Since both of these models were generated using the generalized human reconstruction Recon1 [5], here we use one of the 108 reconstructions generated during the parameter sensitivity analysis to allow for a direct comparison.

As a first step, the size and composition of the different hepatocyte specific reconstructions were compared (Table 1). We find that all three reconstructions have similar size and composition when considering reactions, metabolites, and genes. The mCADRE reconstruction has considerably fewer reactions, the difference stemming mostly from exchange reactions (i.e. the mCADRE reconstruction has 63 fewer reactions than the MBA reconstruction, but 50 fewer exchange reactions). The CORDA reconstruction contains only 6% more reactions than the mCADRE reconstruction and 2.4% more than MBA, which is surprising considering this algorithm does not take a parsimonious approach. This fact is even more significant considering the CORDA reconstructions performed using a single dependency assessment have an average size of 1,828.7 reactions (S1 Text), 3.7% larger than mCADRE and 0.15% larger than MBA, demonstrating the ability of CORDA to perform nearly as concise reconstructions despite not being parsimonious.

**Table 1:** Comparison between CORDA, MBA and mCADRE hepatocyte specific reconstructions.

	MBA	mCADRE	CORDA
**Reactions**	1,826	1,763	1,869
**Exchange reactions**	247	197	249
**Transport reactions**	539	530	618
**Metabolites**	1,360	1,402	1,334
**Unique metabolites**	729	762	722
**Genes**	1,333	1,267	1,242
**Unique genes**	1,051	994	972

The difference in number of reactions between CORDA and other reconstructions stems mostly from a larger number of transport reactions. CORDA contains only 43 more reactions than the MBA reconstruction, but 79 more transport reactions. Similarly, CORDA contains 106 more reactions than the mCADRE reconstruction, but 140 more transport and exchange reactions. This discrepancy indicates that the parsimonious pruning methods are more likely than CORDA to exclude exchange and transport reactions.

**Fig 2:**
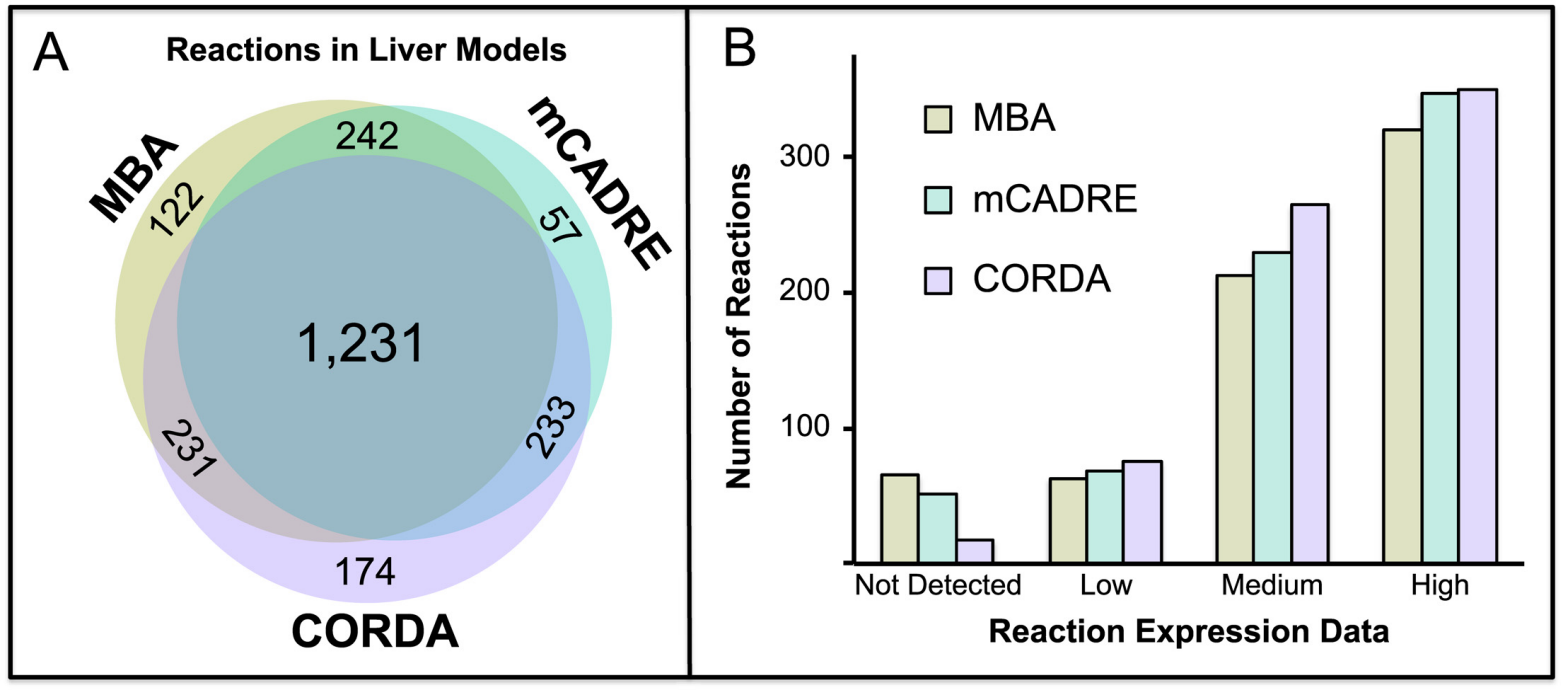
Comparison between MBA, mCADRE and CORDA hepatocyte-specific reconstructions. (A) Venn diagram of reactions included in each model. A total of 1,231 reactions are present in all models. (B) Number of reactions included in each model according to the protein expression data associated with each reaction. The CORDA algorithm includes more low and medium confidence reactions, while including considerably fewer reactions with no protein evidence.

When considering the similarity between models, there are 1,231 reactions present in all three reconstructions, accounting for 67.4% of MBA, 69.8% of mCADRE, and 65.86% of CORDA reactions (Fig 2A). Despite this relatively low overlap, no two models seem to be more similar than the other possible pairs. Furthermore, a higher level of similarity between models is observed when considering unique genes and metabolites, with at least 77% of genes and at least 84% of metabolites in each model shared across all models (S1 Text). Further comparison between the models is available in S1 Text.

Next, the number of HC, MC and NC reactions included in each of the models was analyzed. Here, Not Detected, Low, Medium and High corresponds to the experimental evidence associated with each reaction (Materials and Methods). The CORDA reconstruction showed better agreement with experimental data in all reaction categories (Fig 2B). Particularly, CORDA contained a significantly higher number of medium confidence reactions, 264 as opposed to 229 in mCADRE and 212 in MBA, while including significantly fewer negative confidence reactions, 17 as opposed to 51 in mCADRE and 65 in MBA. It is worth noting that the MBA reactions core was chosen manually and based on data sources different than the one used for the mCADRE and CORDA reconstructions. The difference in reactions core used during the reconstruction process could explain the much lower agreement of MBA with this particular data set.

As a final validation of the CORDA algorithm, the ability of each of the models to perform a series of metabolic tasks was analyzed. These metabolic functions were divided into three categories: (1) amino-acid and ammonium recycling, (2) glucogenic production, and (3) nucleotide production. Briefly, during each test the model was allowed to freely exchange basic metabolites (i.e. water and oxygen), while the remaining exchange reactions were set to mirror the particular test (i.e. uptake of ammonium and release of urea during ammonium recycling test). The model was then forced to produce (1) urea, (2) glucose, or (3) specific nucleotides. If the model was able to do so, the test was considered passed, otherwise, the test was considered failed. If the appropriate exchange reactions were not present to perform the test, the result was considered inconsistent. Further details on how these metabolic tasks were calculated are available in the Materials and Methods section. Test results are summarized in Table 2, and additional information is provided in S1 Text.

**Table 2:** Metabolic test results.

	MBA	mCADRE	CORDA
**Amino-acid recycling**	21/0/0	8/5/8	19/1/1
**Glucogenic**	19/0/0	5/5/9	18/1/0
**Nucleotide production**	4/4/-	8/0/-	6/2/-

Test results are reported as number of tests passed/ tests failed/ and inconsistent test results, meaning the appropriate exchange reactions were not included in the model. Test-specific exchange reactions are not required for nucleotide production tests.

The MBA reconstruction passed all of the amino-acid recycling and glucogenic tests, which is not surprising given the core set of MBA reactions, and therefore the necessary pathways for these tasks, were manually included in the reconstruction. The mCADRE reconstruction passed all eight nucleotide production tests, since these were included in the model building process. Where the tests were not manually included, however, MBA passed only 50% of the nucleotide production tests, and mCADRE passed only 13 of the amino-acid recycling and glucogenic tests, with an additional 17 tests being inconclusive. On the other hand, CORDA passed a total of 43 of the combined 48 metabolic tests (89.6%). This result is significant considering none of these tests were included in the reconstruction process.

Metabolic tests were also performed for all 108 hepatocyte specific reconstructions generated during the parameter sensitivy analysis section. All reconstructions had the same results for all amino-acid recycling, glucogenic, and five of the nucleotide production tests, demonstrating that task results are not heavily dependent on the CORDA parameters or noise (S1 Text). The inability of 39 of the 108 models (36.1%) to produce three of the nucleotides was traced back to the reaction *TRDR*, an MC reaction dependent on the NC reaction *RNDR1*, which was included in some but not all models. This reaction dependency, however, was returned by the CORDA algorithm, and hence the reactions needed to produce all the nucleotides can be easily included upon manual curation.

These analyses demonstrate that the CORDA algorithm provides a reconstruction with better agreement to experimental data and better metabolic functionality when compared to previous, similar methods. Furthermore, the analysis of the 108 models to perform metabolic tasks highlights the importance of the reaction dependencies returned by CORDA in subsequent manual curation, indicating which NC reaction needs to be added back into the model for the desired MC reaction to carry flux.

#### Monte-Carlo sampling

Monte-Carlo Sampling can be used to find a uniform distribution of steady-state flux vectors throughout the metabolic model, providing insight into the shape and size of the model’s solution space [46, 47]. This uniform random sampling technique allows for the unbiased estimation of probability distributions of flux values for each reaction in the model. While the sampled flux values do not necessarily correlate with physiological metabolic fluxes, the sampled distribution can estimate the capability and flexibility of each reaction in the model given the network constraints [46]. This technique has been used to study pathological states in the human red blood cell [46, 47] and mitochondria [48], as well as the interaction between cell types in the human brain [49] and between *M. tuberculosis* and macrophages [25].

Here we performed Monte-Carlo sampling on the CORDA, MBA and mCADRE hepatocyte models, as well as the generalized human reconstruction Recon1. We also introduce a second CORDA reconstruction (CORDA2), calculated using the latest, most up-to-date data from the HPA. To allow for a direct comparison between CORDA and previous algorithms, the CORDA reconstruction used in the previous sections (referred in this section as CORDA1) was calculated using the same data used in the mCADRE reconstruction. This was done to ensure that differences in model functionality, capacity, and differential inclusion of high confidence reactions stemmed from the difference in algorithms used in the reconstruction process, and not from different datasets used to calculate each model. The CORDA2 model, on the other hand, has been calculated to exemplify how the most recent HPA data leads to better model capacity predictions. The protein expression data and reaction groups used in the reconstruction of both CORDA models is available in S1 Table, and both models are available in S1 File. Details of how the flux values were sampled are outlined in the Materials and Methods section.

**Fig 3:**
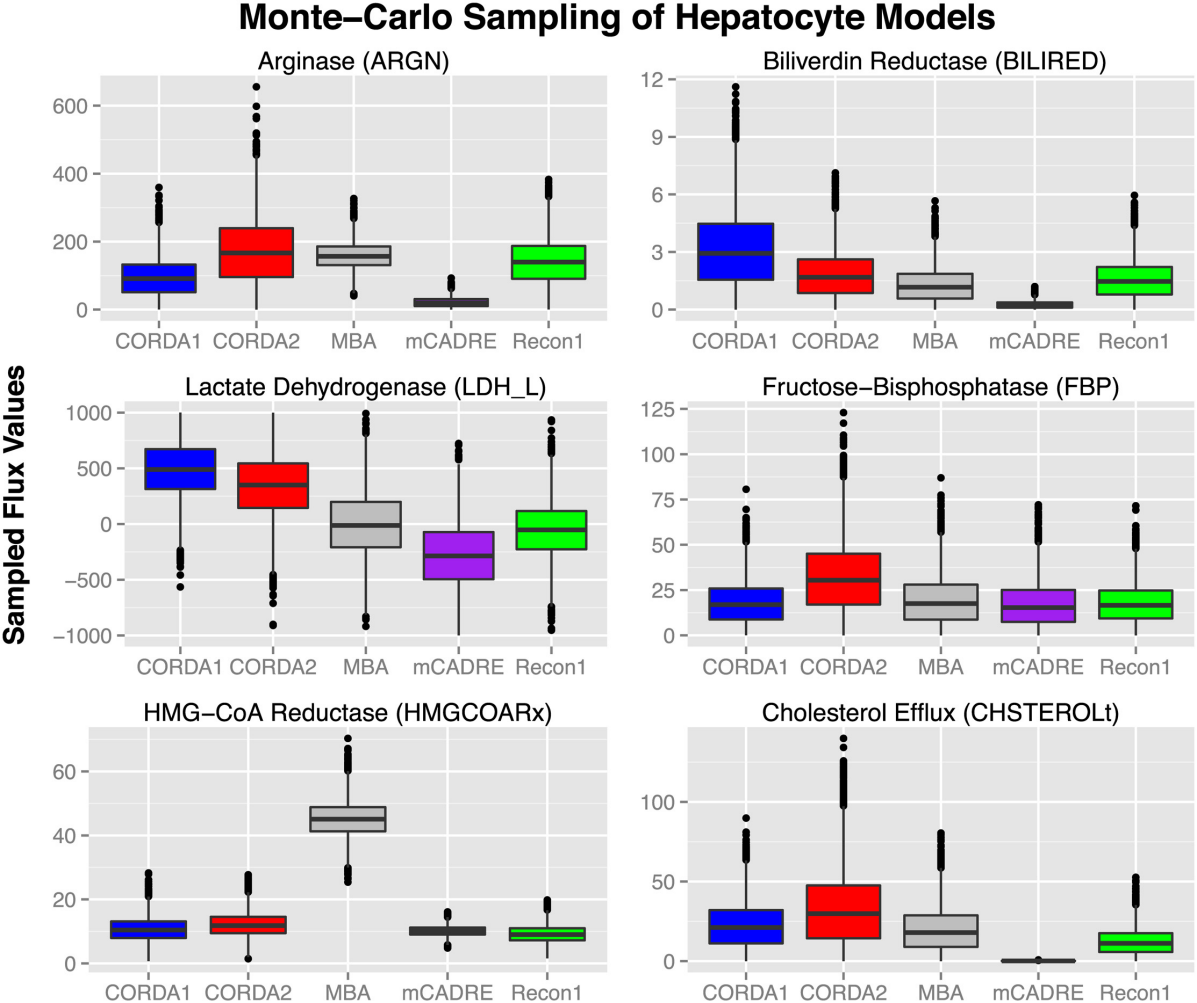
Monte-Carlo sampling of Recon1 and hepatocyte specific models. Distribution of flux values sampled for selected reactions representing hepatocyte specific functions in each of the hepatocyte models and the generalized human reconstruction Recon1. The name of each reaction plotted, as defined in the Recon1 reconstruction, is presented in parenthesis.

The distribution of sampled flux values for reactions representing several hepatocyte specific functions, including production of urea from arginine, production of bilirubin, production of pyruvate from lactate (Cahill cycle), gluconeogenesis, and cholesterol production are plotted in Fig 3. CORDA1 showed a higher capacity than all other reconstructions for bilirubin production and lactate recycling, and a similar capacity for gluconeogenesis and cholesterol efflux. This model only shows a lesser capacity than MBA and Recon1 in the production of urea. Overall, however, these results show that the CORDA algorithm better captures model capability for hepatocyte specific functions when compared to previous models and the generalized human reconstruction. In other words, the subset of Recon1 defined by the CORDA model better represents hepatocyte functions than the subset defined by the MBA and mCADRE reconstructions.

In addition, the sampled fluxes for all reactions considered here showed a significant shift towards higher values in the CORDA2 model when compared to MBA, mCADRE, and the generalized human reconstruction Recon1 (p <10^−20^), including in the production of urea. While CORDA1 showed better functionality in bilirubin production and lactate recycling than CORDA2, CORDA2 outperformed CORDA1 in gluconeogenesis and cholesterol efflux capability. These results suggest that the most recent data from the HPA captures a wider range of tissue-specific functionalities. It is also worth noting that the CORDA models were the only models where the flux through lactate dehydrogenase was highly biased towards positive values, converting lactate to pyruvate (Cahill cycle). All other models considered here showed either an even distribution between positive and negative values, or mostly negative values leading to the production of lactate.

Another interesting result of this analysis is the flux values sampled for HMG-CoA reductase in the MBA reconstruction. This enzyme represents the rate-limiting step in the *de novo* synthesis of cholesterol and other isoprenoids. Flux values sampled for this reaction using the MBA reconstruction are extremely high when compared to other models, and they were never close to or equal to zero. This distribution suggests that this reaction might also be used in other cellular processes, and thus carries a higher flux more frequently during sampling. To investigate this possibility, we analyzed which reactions are dependent on the HMG-CoA reductase reaction (*HMGCOARx*) in each of the hepatocyte models. This was done by evaluating which reactions lose their ability to carry flux upon setting the upper and lower bounds of *HMGCOARx* to zero. While in the CORDA models only reactions in the cholesterol metabolism, endoplasmic reticulum transport, and peroxisomal transport pathways are blocked, a much larger number of reactions are blocked in the MBA and mCADRE reconstructions. In both of these models, a high number of bile acid biosynthesis reactions lose functionality upon blockage of *HMGCOARx*. Reactions in other sub-systems, such as Lysine metabolism and purine catabolism in MBA, and taurine and hypotaurine metabolism, vitamin D metabolism, and CoA biosynthesis in mCADRE, also have a loss of function (S1 Text). Overall, upon blockage of *HMGCOARx*, 188 additional reactions in the MBA model, and 156 in the mCADRE model lose function, compared to 30 in CORDA1 and 33 in the CORDA2 model (S1 Text). This analysis demonstrates how parsimonious algorithms are likely to remove alternative pathways from the model, conferring very high levels of influence over the network to particular reactions.

### Generation of multiple healthy and cancer tissue-specific models

Following the algorithm validation, we generated a library of 76 healthy and 20 cancer tissue-specific models using CORDA. In order to generate the most comprehensive models possible, we used the generalized human reconstruction Recon2 [9] in the calculation of this library. Recon2 is one of the most comprehensive human reconstructions performed to date, containing approximately twice the amount of reactions than Recon1, 1.7 times more unique metabolites, and 1.2 times more unique genes. Details of how the reconstructions were calculated can be found in the Materials and Methods section.

#### Identification of essential metabolites

As an initial validation of this library, we analyzed the reconstructions for the identification of essential metabolites specific to cancer. Essential metabolites are necessary to carry out specific cellular functions, and can be used for the identification of possible antimetabolites. Antimetabolites, in turn, are structurally similar to essential metabolites but cannot be used by the cell, thus stalling enzymes consuming the essential metabolite through competitive inhibition [50]. By targeting cellular functions specific to cancer, antimetabolites have been widely used in the treatment of multiple types of cancer [51–53].

The identification of essential metabolites can be predicted computationally using GEMs [15, 16, 54]. Given a metabolite to be tested, all reactions consuming that metabolite are blocked in the forward direction, and reactions producing the metabolite are blocked in the backwards direction. An array of essential metabolic functions is then tested, and failure to complete any of those functions renders the metabolite essential for that GEM. All 76 healthy and 20 cancer specific reconstructions calculated here were tested for all 271 unique metabolites present in all models, using the 32 metabolic functions included in the reconstruction process (S1 Table). Two metabolites selectively targeted cancer over healthy reconstructions: phosphatidylethanolamine (*pe_hs*), essential in 1.3% of healthy and 10% of cancer specific reconstructions, and triglyceride (*tag_hs*), essential in 31.6% of healthy and 70% of cancer specific reconstructions. Both of these metabolites are involved in fatty-acid and glycerophospholipid pathways, which have been previously identified as specifically essential to cancer tissues [15, 16].

#### Composition analysis of cancer and healthy tissue models

**Fig 4:**
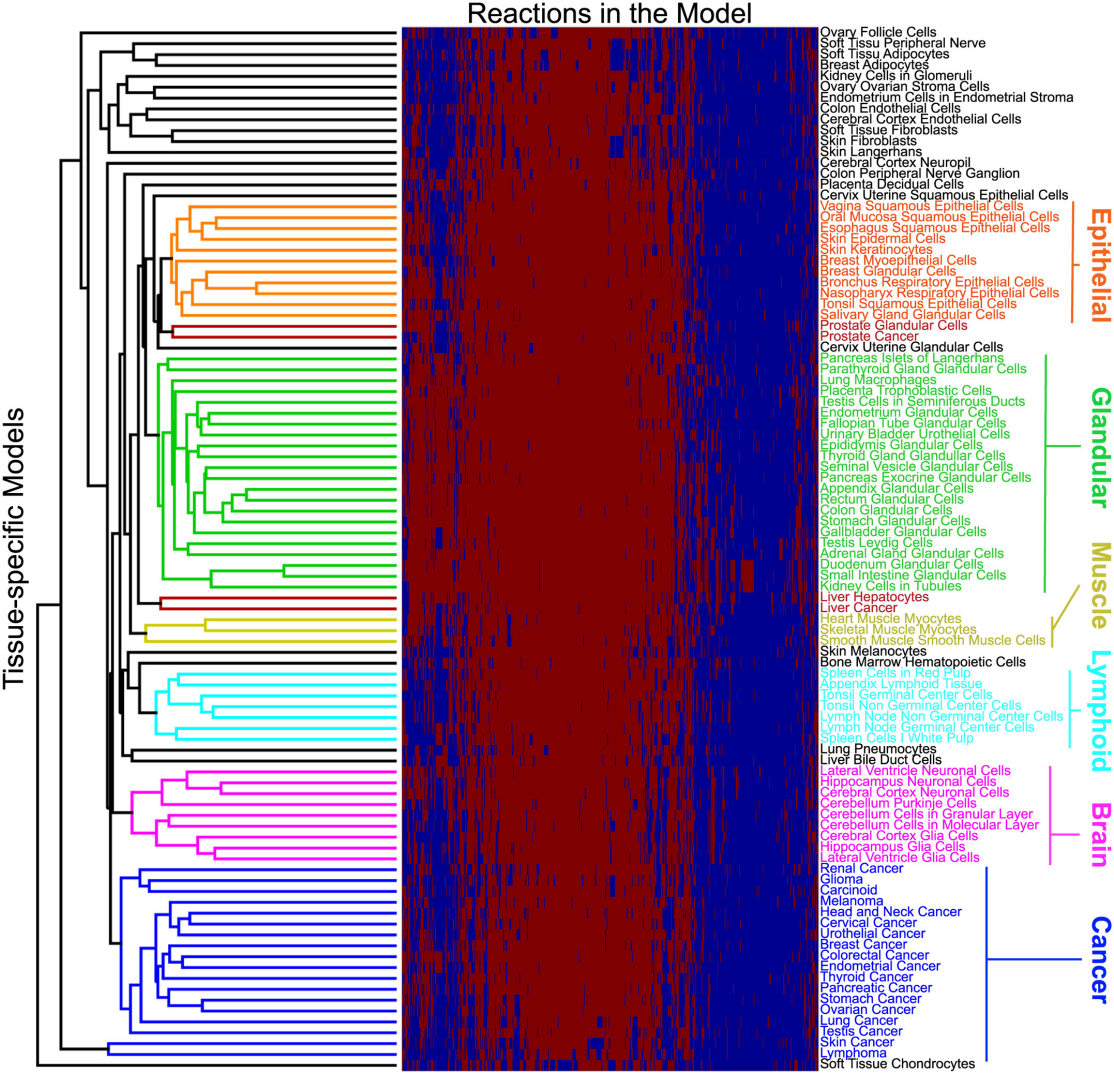
Clustering of tissue specific models. Healthy- and cancer-specific models clustered according to reactions present in each model. Highlighted clusters indicate epithelial and myoepithelial like tissues (orange), glandular like tissues (green), muscle tissues (yellow), lymphoid like tissues (cyan), brain cells (magenta), cancer tissues (blue), and cancers clustered with their healthy counterpart (red). Red squares indicate reactions present in the model and blue squares indicate reactions that are absent.

Next, the 96 tissue-specific models were clustered according to the reactions present in each of them (Materials and Methods). The clustering results are summarized in Fig 4. We find that the tissue-specific models largely cluster according to tissue type, with most of the cancer models clustering together. The only exception stems from the liver and prostate cancer models, which cluster with their healthy counterparts (Fig 4).

**Fig 5:**
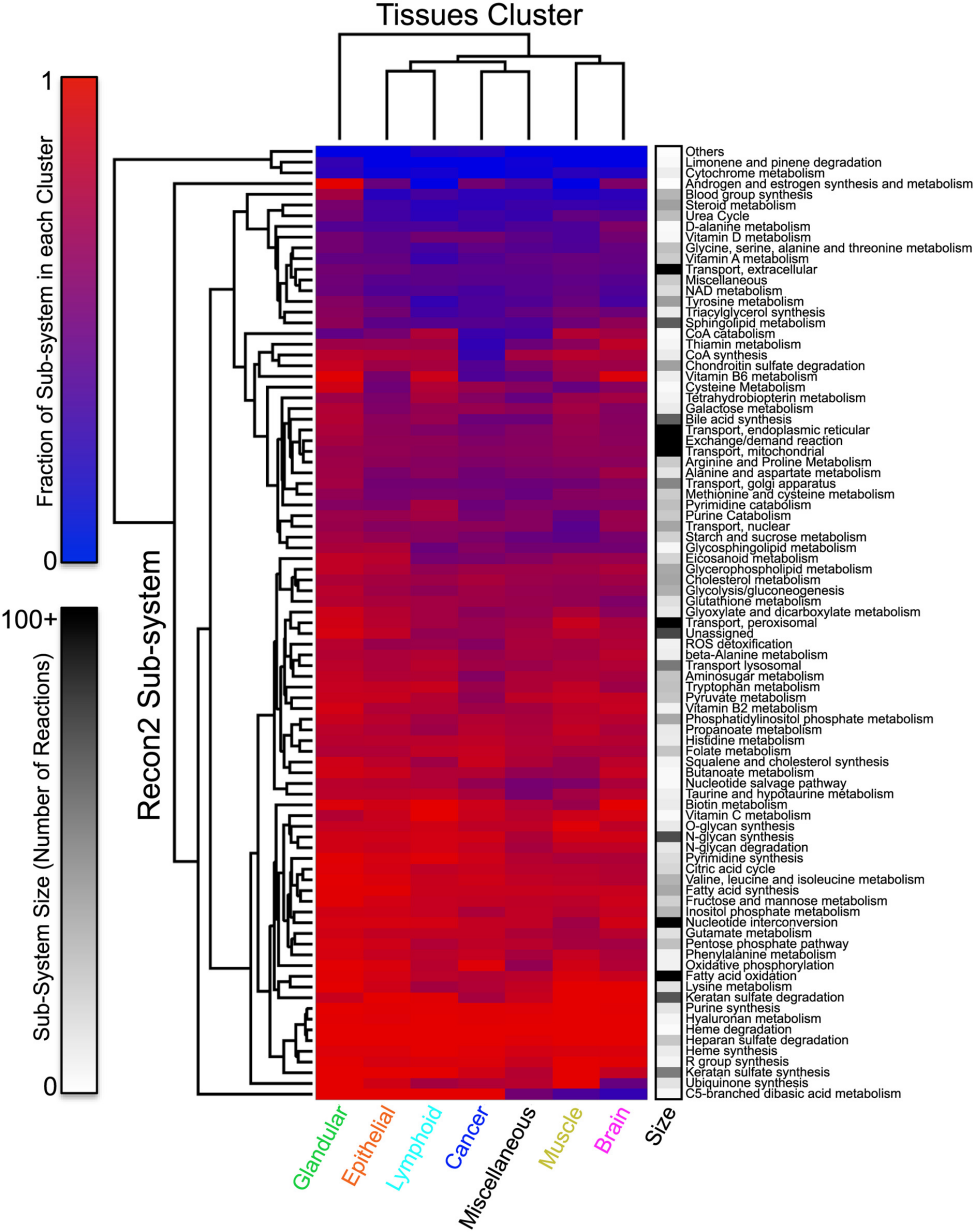
Clustering of subsystems for each model category. The presence of reactions in each subsystem, for each model category, was calculated and clustered. Heat map represents the fraction of reactions in each subsystem included on average in models of the specific category. Number of reactions in each subsystem considered is also included.

Following the clustering results described above, we divided the models into seven categories according to the results presented in Fig 4. These categories are glandular (green), epithelial (orange), lymphoid (cyan), cancer (blue), muscle (yellow), brain (magenta) and miscellaneous (black), which includes all of the remaining models. The presence of reactions in 89 different subsystems was then calculated and clustered for each of the model categories (Materials and Methods). Results are summarized in Fig 5. Evidence for the up- or down-regulation of many metabolic pathways differentially included or excluded from cancer models as opposed to healthy tissues is available in the literature [55–82] and highlighted in the discussion section. Overall, this clustering analysis further confirms the ability of CORDA to generate reconstructions in agreement with experimental data, and validates the library of healthy and cancer tissue reconstructions generated here.

Single reactions that are present most often in cancer but not in healthy, and in healthy but not in cancer tissue-specific reconstructions, were also analyzed (Table 3). All reactions that are present most often in cancer tissues have been shown to be up-regulated in at least one type of cancer. Reactions that are not gene-associated are part of sarcosine or folate metabolism pathways, both of which have been shown to be up-regulated in cancer [70–72, 83, 84]. Similarly, reactions that are present most often in healthy tissue reconstructions, but not cancer, have largely been shown to be down-regulated in cancer (Table 3). Many of these reactions are part of D-glucosamine and n-3 polyunsaturated fatty acids metabolisms, both of which have been shown to selectively target and kill cancer cells [58–61, 85–87]. An additional three reactions, *PNTK*, *PPCDC* and *PPNCL3* are part of coenzyme-A synthesis pathways, a pathway also implicated as significantly excluded from cancer models during the clustering analysis (Fig 5). These reactions are largely excluded from cancer reconstructions due to the gene *PPCS*, the only gene from these three reactions with supportive information in the HPA, and thus included in the reconstruction process. *PPCS* is expressed in all healthy tissues, with medium or high confidence in 33 of them, but is largely low or negatively expressed in most cancer tissues. Similarly, the reaction *ACOAO7p*, dependent on the gene *ACOX1*, was largely included in healthy tissues and not cancer tissues according to experimental data.

**Table 3:** Reactions differentially included in healthy versus cancer tissue models.

Reaction	Subsystem	Associated Genes	Cancer Models	Healthy Models	Literature Evidence
**Reactions present in cancer tissue models most often:**					
*DNDPt15m*	Transport, mitochondrial	60386	95%	32.9%	Breast Cancer [88]
*EX_sarcs(e)*	Exchange/demand reaction	-	85%	22.4%	Prostate Cancer [83, 84]
*H2O2syn*	Tyrosine metabolism	53905, 50506	90%	39.5%	Prostate Cancer [89]
*MTHFD2m*	Folate metabolism	10797	65%	7.9%	Multiple [71, 73], Breast [70, 72]
*SARCStex*	Transport, extracellular	-	85%	22.4%	Prostate Cancer [83, 84]
*r0514*	Folate metabolism	1719	85%	10.5%	Multiple [71, 90]
*r0961*	Transport, extracellular	1356	70%	9.2%	Multiple [91, 92]
*r0962*	Transport, mitochondrial	-	85%	10.5%	Multiple [71], Breast [70, 72]
*r2085*	Transport, extracellular	6566	70%	9.2%	Neuroblastoma [93]
*r2086*	Transport, extracellular	6566	90%	35.5%	Neuroblastoma [93]
**Reactions present in healthy tissue models most often:**					
*ACOAO7p*	Fatty acid oxidation	51	15%	97.4%	-
*ALCD22_D*	Pyruvate metabolism	124–128, 130, 131, 137872, 284273	25%	97.4%	Colorectal Cancer [94, 95]
*DADA*	Nucleotide interconversion	100	5%	86.8%	Multiple [96], Renal adenocarcinoma [97], Gastric [98]
*DADAe*	Nucleotide interconversion	100	5%	86.8%	Multiple [96], Renal adenocarcinoma [97], Gastric [98]
*DESAT24_1*	Fatty acid synthesis	9415	5%	80.3%	Multiple [99]
*EX_gam(e)*	Exchange/demand reaction	-	20%	94.7%	Multiple [58–61]
*EX_tethex3(e)*	Exchange/demand reaction	-	5%	76.3%	Multiple [85–87]
*EX_tetpent3(e)*	Exchange/demand reaction	-	5%	80.3%	Multiple [85–87]
*FACOAL245_2*	Fatty acid oxidation	2180	5%	80.3%	Hepatoma [100], Lung [101], Colon [102]
*FACOAL246_1*	Fatty acid oxidation	2180	5%	76.3%	Hepatoma [100], Lung [101], Colon [102]
*GAMt1r*	Transport, extracellular	6513, 6514, 6517	20%	94.7%	Multiple [58–61]
*HEX10*	Aminosugar metabolism	2645, 3098, 3099, 3101, 80201	20%	94.7%	Multiple [58–61]
*O2Stx*	Transport, peroxisomal	-	25%	98.7%	-
*PNTK*	CoA synthesis	53354, 55229, 79646, 80025	20%	98.7%	-
*PPCDC*	CoA synthesis	60490	20%	98.7%	-
*PPDOx*	Pyruvate metabolism	8574	20%	97.4%	Hepatocellular Carcinoma [103]
*PPNCL3*	CoA synthesis	79717	20%	98.7%	-
*SPODMx*	ROS detoxification	6647	25%	98.7%	Multiple [104]
*TETHEX3t*	Transport, extracellular	-	5%	76.4%	Multiple [85–87]
*TETPENT3t*	Transport, extracellular	-	5%	80.3%	Multiple [85–87]

References for reactions largely present in cancer models refer to up-regulation in cancer metabolism, while references for reactions largely present in healthy tissue models refer to their down regulation in cancer cells.

Finally, reactions that are present in three or fewer healthy models, and reactions that are present in a single cancer model, were analyzed in order to assess CORDA’s ability to include tissue specific reactions (S1 Table). We found that the inclusion of tissue specific reactions are largely aligned with our knowledge of metabolism. Bile acid and urea cycle reactions are concentrated on the hepatocyte and intestinal mucosa reconstructions [105]; tyrosine metabolism, a precursor to melanin, is highly active in skin melanocytes and epidermal models; the soft tissue adipocyte model contains a specific vitamin D metabolic reaction, a pathway highly associated with this tissue [106, 107]; steroid metabolism reactions are concentrated in adrenal, duodenum and prostate glandular, as well as testis Leydig cell reconstructions; and the iodine exchange reaction is unique to the thyroid gland model. Similarly, bile acid synthesis and cholesterol metabolic reactions are highly concentrated in the liver cancer reconstruction; chondroitin sulfate degradation reactions are highly concentrated in the prostate cancer model [108–111]; and tyrosine metabolism reactions are largely present in the thyroid [112, 113] and melanoma [114] cancer reconstructions (S1 Table).

#### Monte-Carlo sampling of cancer and healthy tissue models

**Fig 6:**
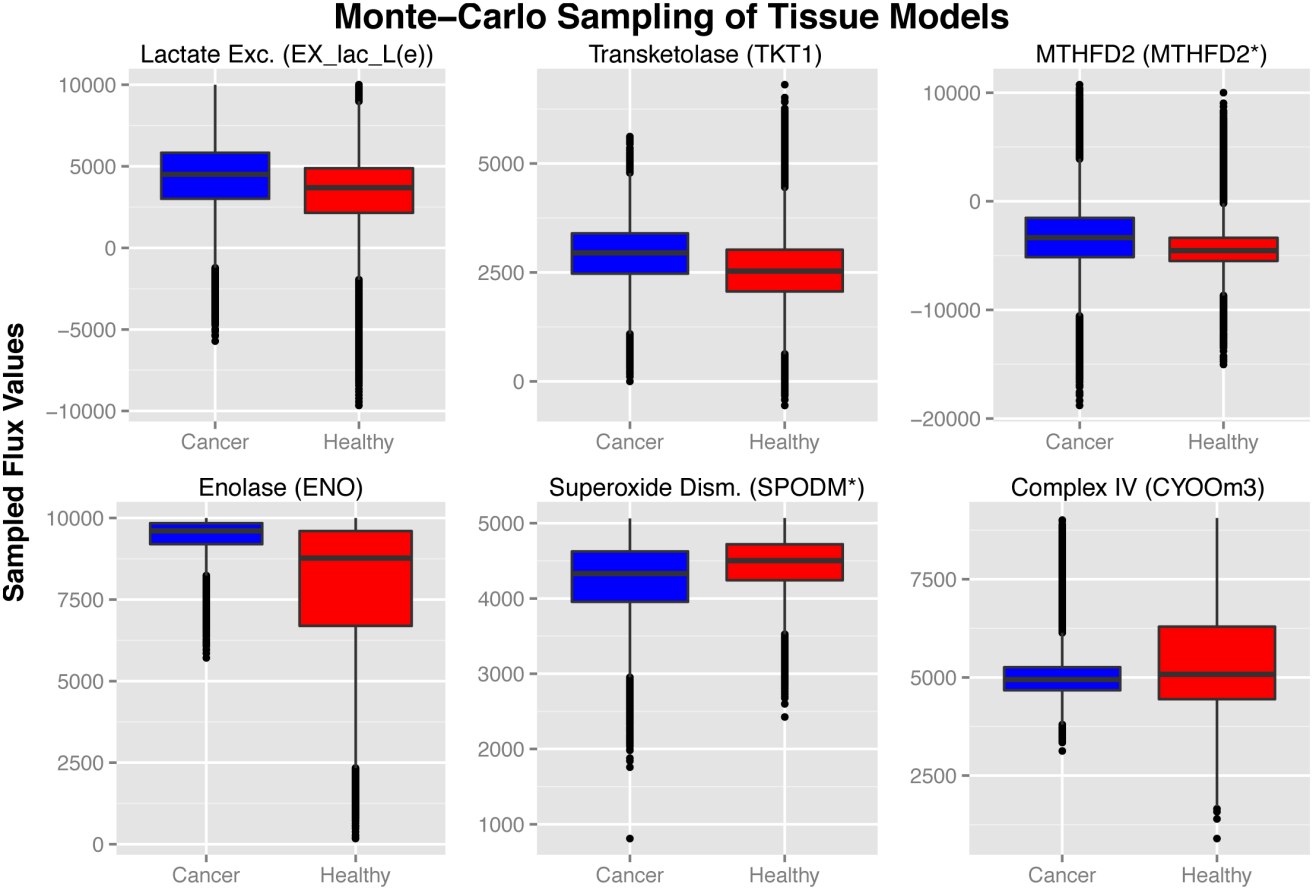
Monte-Carlo sampling of cancer and healthy tissue models. Flux values sampled from all cancer and healthy tissue models. Recon2 reactions plotted in each boxplot are indicated in parenthesis. Asterisk indicates groups of reactions taking place in different cellular compartments. According to general cancer metabolism, cancer models showed a higher capacity through lactate production, pentose phosphate pathway, MTHFD2, and glycine hydroxymethyltransferase, while showing a lower capacity through oxidative phosphorylation and superoxide dismutase.

Monte-Carlo Sampling was also performed on each of the 96 cancer and healthy tissue models. Due to the heterogeneity between cell types, flux values were analyzed between all cancer and healthy tissue models together (Materials and Methods). The distribution of sampled flux values for selected reactions or group of reactions are plotted in Fig 6. Plots for individual healthy and cancer models are available in S1 Text. Similar to the hepatocyte specific analysis, the sampled values showed good agreement with experimental data and our understanding of cancer metabolism. Cancer models showed an overall greater capacity for lactate secretion (in accordance with the Warburg effect [115, 116]), glycolysis [71], the pentose phosphate pathway [116] (particularly through the up-regulation of TKT1 [71]), and through methylene-THF dehydrogenase (MTHFD2) in the conversion of 5,10-methylene-THF to 5,10-methenyl-THF (positive direction)[71]. On the other hand, pathways that have been shown to be down-regulated in cancer demonstrate a decreased capacity in cancer models when compared to healthy tissue models, including mitochondrial respiration (Complex IV)[115] and superoxide dismutase [71, 104].

These results demonstrate the ability of Monte Carlo sampling to predict the capacity of cancer versus healthy tissue models beyond simple topological analysis (i.e., simply looking at the presence or absence of certain reactions or pathways in the model). For instance, this analysis is able to capture a higher capability of cancer models for lactate secretion, and a lower capacity through superoxide dismutase, based on model topology alone, even though these reactions are present in all the models in the library. This analysis is made possible by the faster, high throughput capability of the CORDA algorithm, allowing for the generation of a library of tissue-specific models based solely on experimental data. While mCADRE allows for a relatively fast and high throughput generation of tissue-specific models, results shown in Fig 3 suggest that these reconstructions show poor functionality predictions. MBA, on the other hand, yields much better predictions of model functionality than mCADRE, but still falls short of the CORDA reconstructions according to our hepatocyte specific analysis (Fig 3). The computational cost of the MBA algorithm also renders the generation of a library of models extremely expensive and time consuming, especially if the core set of reactions were to be selected manually. In sum, CORDA resulted in direct comparisons of cancer versus healthy tissue metabolism, as well as more accurate reconstructions of hepatocyte function compared to prior tissue-specific metabolic models, in a computationally efficient manner.

## Discussion

Here we introduced a novel tissue-specific algorithm based on Cost Optimization Reaction Dependency Assessment (CORDA). CORDA relies solely on FBA, rendering it more computationally efficient than previous methods. CORDA takes a non-parsimonious approach to the reconstruction process, based on the addition of valuable reactions to the reconstruction as opposed to the removal of non-essential reactions. We showed that the CORDA algorithm provides reconstructions that agree better with experimental data, and that demonstrate better metabolic functionality than prior methods like MBA and mCADRE. Furthermore, CORDA provides reaction associations that can greatly assist subsequent manual curation, while maintaining the reconstructions only slightly larger than previous parsimonious approaches. Monte-Carlo sampling analysis also demonstrates that the CORDA generated models provide better predictions of tissue-specific functionality.

In addition to the algorithm validation, we generated a library of 76 healthy and 20 cancer tissue-specific reconstructions, which show considerable agreement with our current knowledge of healthy tissue and cancer metabolism. First, as an initial validation of our cancer and healthy tissue models, we computationally predicted metabolites that are more frequently essential in cancer models than healthy tissues [15, 16, 54]. Two metabolites were implicated in this analysis: phosphatidylethanolamine (*pe_hs*) and triglyceride (*tag_hs*), both of which are part of metabolic pathways previously implicated as cancer specific [15, 16]. While future work is merited to identify more specific essential metabolites (e.g. through the inclusion of more comprehensive metabolic tasks in the tissue reconstruction process, and more metabolites in the essential metabolite identification algorithm), these results help validate the cancer and healthy tissue reconstructions presented here.

Following this analysis, we demonstrated that the tissue models calculated by CORDA cluster largely according to tissue type. Similar clustering patterns, based on gene expression and proteomics data, have been observed experimentally. In particular, based on the expression of over 30,000 genes across multiple individuals and tissues, one study found that brain, muscle, and liver tissues, as well as Epstein-Barr virus-transformed lymphocytes, form well defined groups, while skin, adipocytes, and nerve tissues cluster closely together [117]. A separate study used in the generation of the HPA, based on protein evidence from almost 17,000 protein-coding genes in 44 major tissues and organs, also showed that tonsils, spleen, appendix, and lymph node tissues cluster closely together, and that bone marrow clusters separately, but close to these lymphoid tissues [45].

Evidence supporting many of the apparent exceptions identified by our clustering analysis is also available. For instance, Uhlén et. al. found that brain and liver tissues, along with testis, cluster considerably separate from other tissues and closer to each other, which is what we observed by clustering the CORDA models. The same study found that prostate tissue clusters closely with salivary glands [45]. It is worth noting that good agreement with the data by Uhlén et. al. is expected, given that a subset of this data was used to generate the tissue-specific models. This agreement, however, suggests that the similarities between tissues shown by Uhlén et. al.[45] and Melé et. al.[117] at the gene expression and protein level are also present in the metabolic enzymes level.

Additionally, breast and salivary glands are known to share many morphological features, and studies have shown that both can give rise to tumors with similar morphology [118, 119] and myoepithelial differentiation [120]. These finding can explain why breast and salivary glands clustered with epithelial and myoepithelial cells, as opposed to glandular cells. Finally, skin cancer and non-Hodgkin’s lymphoma appear frequently as secondary cancers in immunosuppressed individuals [121, 122]. This could lead to cancers with significantly different metabolic profiles, supporting their separation from the remaining cancer models.

Clustering of tissue-specific models according to subsystems has also highlighted many differences between healthy and cancerous tissues at the pathway level (Fig 5). Evidence for many of these differences are also available in the literature, including:

- **Thiamine metabolism:** Thiamine deficiency is common in advanced cancer patients [55, 56]. Thiamine supplementation has been shown to increase tumor proliferation *in vitro* through transketolase activation [55], and multiple studies have linked thiamine metabolism to cancer through numerous mechanisms [57].
- **Aminosugar metabolism:** D-glucosamine, the most abundant aminosugar and an important precursor in many biological pathways, has been shown to reduce tumor proliferation [58]. Although the mechanism of this inhibition is unknown, studies have suggested it may be through the targeting of cellular membranes [59], protein synthesis through p70S6K regulation [60], or protein N-glycosylation [61].
- **Pyruvate metabolism:** Pyruvate connects many metabolic pathways, and alterations in these pathways play an important role in cancer metabolism [62, 63]. Recent studies have shown that mitochondrial pyruvate carrier function is lost in cancer, and low expression of this protein is associated with poor survival [64]. This loss of function was also shown to induce the Warburg effect, a hallmark of cancer [64]. Additionally, the pyruvate kinase isoenzyme PKM2 has been shown to play a key role in cancer metabolism, diverting glucose to anaerobic pathways [65].
- **Glycosaminoglycans degradation:** The accumulation of glycosaminoglycans in cancer cells has been widely established, and therapies targeting these polysaccharides have been proposed [66, 67]. In particular, chondroitin sulfate proteoglycans have been shown to accumulate in cancer cells and promote tumorigenesis by interacting with surface receptors [68]. Furthermore, keratan sulfate has been shown to accumulate in the serum of patients with cartilage tumors, and serum levels were shown to decrease upon tumor removal [69]. Our results suggest that this accumulation is coupled with a down-regulation of glycosaminoglycans degradation.
- **Folate Metabolism:** Folate metabolism, in particular the enzyme methylenetetrahydrofolate dehydrogenase (MTHFD2), has been shown to be up-regulated in cancer cells [70–73], and it has been shown to contribute to energy and purine requirements in cancer [73]. Folate pathways have also been associated with poor prognosis [71, 72] and increased cell proliferation *in vitro*[73]. Also, anti-folate agents have been shown to reduce cancer proliferation, and have been proposed as anti-cancer agents [73, 74]. Monte-Carlo sampling analysis has also shown a more positive flux distribution through the reactions *MTHFD2* and *MTHFD2r* in cancer over healthy tissue models (Fig 6), in accordance with our knowledge of cancer metabolism [71].
- **Squalene and cholesterol synthesis:** Squalene is an important precursor of cholesterol. Squalene synthase expression has been shown to be increased in prostate cancer [76], and inhibition of this enzyme has been demonstrated to cause cell death in prostate cancer [75, 76]. Furthermore, squalene oxidase has been indicated as an oncogene in both pancreatic [77] and breast [78] cancer.
- **Oxidative phosphorylation:** Oxidative phosphorylation pathways have long been considered down-regulated in cancer tissues. Recent studies have found, however, that these pathways remain intact [79] or even increase during metastasis [80–82]. The activity of this pathway in cancer versus healthy tissue models is further discussed during the Monte-Carlo sampling analysis discussion.

Single reactions included most often in cancer or healthy tissue models were also analyzed, and again literature evidence has been found to support many of them (Table 3). Two surprising findings stemmed from this analysis. First is the predicted down-regulation of CoA synthesis reactions, implicated in both the subsystem and single reaction analyses. Upon further inspection, we traced this differential inclusion to the gene *PPCS*, the only gene related to this pathway included in the reconstruction process, which is significantly down-regulated in cancer cells [44, 45]. Second, the exclusion of *ACOAO7p* from most cancer models is also unexpected, since this reaction is part of the fatty-acid oxidation pathway, which has been shown to be up-regulated in cancer tissues [123, 124]. Protein evidence of this reaction’s associated gene, *ACOX1*, supports this exclusion from cancer models [44, 45], suggesting an alternate pathway for palmitoyl-CoA oxidation in cancer tissues.

Finally, Monte-Carlo sampling was also performed in all healthy and cancer tissue models. Sampling results demonstrate that cancer models show an increased capacity through pathways that are largely up-regulated in cancer metabolism, and a reduced capacity through pathways previously shown to be down-regulated. Interestingly, mitochondrial respiration showed a slightly reduced and tightly constrained capacity in cancer over healthy tissue models, despite the presence of a larger number of oxidative phosphorylation reactions in cancer models (Fig 5). For decades, the role of mitochondrial respiration was thought to be decreased in cancer tissues due to their high glycolytic capacity. In recent years, however, researchers have shown that this pathway actually plays an important role in cancer metabolism [125, 126]. Our results suggest that although a larger number of oxidative phosphorylation reactions are present in cancer models, the activity of this pathway is tightly regulated by cancer metabolism topology (Fig 6). On one hand, the low probability of cancer models reaching high cytochrome c oxidase flux values compared to healthy tissues is in line with cancer’s high glycolytic potential. At the other extreme, the low probability of cancer models reaching relatively low cytochrome c oxidase sampled fluxes is in line with the key role played by mitochondrial respiration in cancer metabolism uncovered in recent years.

We have also investigated the differences in glycine hydroxymethyltransferase capacity in cancer versus healthy tissue models (S1 Text). This reaction is dependent on two proteins, *SHMT1* and *SHMT2*, which correspond the cytosolic and mitochondrial isozymes respectively. Both these proteins have been shown to be up-regulated in cancer over healthy tissue models [127], although *SHMT2* has been so to a greater extent [71, 127]. The over expression of these proteins, however, has been shown to be heavily dependent on cancer type [127]. This claim is supported by the protein expression of *SHMT2* in the HPA, where half the cancer types considered have samples with both high and not detected *SHMT2* expression. This variability could explain why the distribution of reactions associated with these genes is similar between cancer and healthy tissue models (S1 Text). Some cancer types, however, show a considerable increase in *SHMT2* expression when compared to their healthy counterparts, including breast, glioma, head and neck, lung, stomach, testicular, and thyroid cancer. In all but one of these models (glioma), the flux distribution of glycine hydroxymethyltransferase was shown to be considerably shifted towards higher values when compared to their healthy counterparts (S1 Text). These results demonstrate CORDA’s ability to predict cancer type specific functionality, and not only differences between all cancer and healthy tissues taken together.

The CORDA tissue-specific reconstruction algorithm, as well as the healthy and cancer tissue-specific reconstructions presented here, introduce a new approach for the development of comprehensive tissue-specific metabolic reconstructions. These reconstructions can generate novel insights into both healthy and diseased human metabolic behavior. Furthermore, the ability of CORDA to generate models based solely on experimental data, along with the computational efficiency of this algorithm, allows for continuous updates of this library of tissue-specific models, both as more experimental data is updated and made available, and as more comprehensive human metabolic reconstructions are developed.

## Materials and Methods

### The Cost Optimization Reaction Dependency Assessment (CORDA) algorithm

While previous methods determined reaction dependencies using Flux Variability Analysis (FVA), the CORDA algorithm takes a different approach, referred here as *dependency assessment*. The novelty of this method lies not in the LP formulation itself, which is the same as the widely established Flux Balance Analysis (FBA), but in the model modifications performed prior to the application of FBA, as well as the interpretation of the flux distribution returned. Assuming we want to test whether a given reaction, *x*, is dependent on the presence of a group of reactions, *Y*, to carry flux, CORDA proceeds in five steps. The parameters required for the CORDA algorithm are summarized in Table 4:

1. As a first step, *x* is constrained to carry a strictly positive or strictly negative flux ±*ϵ*, depending on whether the reaction is reversible or not, and which direction we wish to test. The magnitude of *ϵ* can be arbitrarily small.
2. Each reaction in *Y* is associated with a high “cost” *γ*. This cost is added to each reaction as a pseudo metabolite, while splitting reversible reactions into forward and backwards reactions. That is, a reaction “*A* ⇔ *B*” is split into “*A* ⇒ *B*+*cost*” and “*B* ⇒ *A*+*cost*”. This way, the cost is positively produced whether the reaction is taking place in the forward or backwards direction. At this step, reactions not in *Y* are assigned a cost of zero.
3. Each reaction cost is increased by a small random value sampled uniformly between zero and *κ*. The parameter *κ* is several orders of magnitude lower than *γ*. This noise is added to account for multiple pathways which allow *x* to carry a flux *ϵ*, and are associated with the same cost. With the added noise, these pathways will have slightly different costs.
4. A reaction consuming this pseudo metabolite is added to the reconstruction and set as the model objective. This added reaction is then the only mean of cost consumption, while all other model reactions produce the pseudo metabolite regardless of directionality.
5. FBA is performed while minimizing the flux through the cost consuming reaction. The flux distribution obtained is then the flux distribution with minimal cost needed to maintain the strictly positive or strictly negative flux ±*ϵ* of *x*. Any reaction in *Y* predicted to carry a flux in this flux distribution will be referred to as *associated* with *x*.

**Table 4:** Parameters required for the dependency assessment and CORDA tissue-building algorithm.

Parameter	Parameter Use
*γ*	Cost associated with undesirable reactions (*Y*).
*κ*	Order of magnitude of noise added to reaction costs. Each reaction cost is increased by a value sampled uniformly between zero and *κ*.
*ϵ*	Magnitude of flux value by which the reaction being tested (*x*) is constrained.
*n*	Number of times each reaction dependency is assessed in order to sample from pathways with equivalent costs.
*p*	Threshold for the inclusion of Negative Confidence reactions in step 2 of the tissue-specific algorithm.

It is worth noting that the high cost reactions implicated in step five are not necessarily essential for *x* to carry a flux ±*ϵ*, but are the set of reactions in *Y* that combined carry the minimal amount of flux. That is, no flux distribution through the metabolic network allows for the predefined flux through *x* with a lower combined flux through the reactions of *Y*. For instance, if one of the reactions in *Y* deemed associated with *x* were to be removed from the reconstruction, *x* could still be able to carry a flux ±*ϵ*, but the combined flux through the reactions in *Y* would be larger than before. This way, this dependency assessment does not minimize the number of undesirable reactions to allow *x* to carry flux, but instead the combined flux through them. Naturally, however, a lower number of reactions would more easily allow for a lower combined flux. It is also for this reason that throughout the manuscript we use the term *associate* instead of *dependent*. Throughout the literature, referring to one reaction as *dependent* on another means the removal of the later from the model negates the former’s ability to carry flux, which is not necessarily the case for the reaction *associations* defined here.

Another significant advantage of this dependency assessment over previous pruning algorithms is that it requires only the LP problem solved during FBA, rendering it much faster than previous methods. While MBA and mCADRE used a much faster variation of FVA, it is still considerably more computationally expensive than LP. Although mCADRE is up to three orders of magnitude faster than MBA [30], the mCADRE model used in this study took about 4 hours to be calculated in a 2.34 GHz CPU with 4G RAM using the IBM CPLEX solver [30]. The CORDA reconstruction, on the other hand, using the same data and general human reconstruction, took under 30 minutes in a 2.66 GHz CPU with 4G RAM using the Gurobi solver [128].

In order to obtain a tissue-specific metabolic reconstruction using this dependency assessment, we define the Cost Optimization Reaction Dependency Assessment (CORDA) algorithm. This algorithm takes as input the reactions in the generalized human reconstruction divided into four categories:

- **High Confidence (HC) reactions:** Reactions that are sure to be included in the tissue-specific reconstruction.
- **Medium Confidence (MC) reactions:** These reactions will be included in the final reconstruction if they are not dependent on negative confidence reactions associated with few MC reactions.
- **Negative Confidence (NC) reactions:** Reactions not to be included in the final tissue-specific reconstruction. These will only be included if they are associated with any HC or a high number of MC reactions. The NC and MC reactions core is then flexible, while the HC core is not.
- **Other (OT) reactions:** All remaining reactions in the generalized human reconstruction not included in the HC, MC or NC groups.

Here, we also allow for the inclusion of metabolic tasks in the HC group. That is, during the CORDA algorithm, sinks can be specified for given metabolites, and added to the model when tested to ensure the final tissue model can produce these metabolites. These reactions are added when being tested then immediately removed from the model, so that none of these metabolic task reactions are present when other reactions are being tested, and no two test reactions are present in the model at the same time. The 32 metabolic tasks included in all CORDA reconstructions in this manuscript are available in S1 Table.

While the definition of these reaction groups can be left to the user’s discretion, here we defined the four groups according to proteomics data from the HPA [44, 45], and boolean gene-reaction rules included in the generalized reconstructions Recon1 and Recon2. In the HPA, each protein is classified as being Not Detected, or present at Low, Medium or High levels in each tissue. The gene-reaction association rules are composed of gene names and “AND” and “OR” boolean associations. For instance, the reaction *r0634* in Recon2 has the boolean rule “*HADHB AND (ACAA2 OR ACAA1)*”, and can therefore be considered active if the gene HADHB, as well as ACAA2 or ACAA1, are active.

Using this boolean mapping, gene IDs were first replaced by the numerical values -1, 1, 2, and 3, corresponding to Not detected, Low, Medium and High protein expression levels respectively. Genes not included in the dataset were assigned a numerical value of zero. Next, AND boolean associations were replaced by the function MIN; OR boolean associations were replaced by the function MAX; and the expression was evaluated. Reactions with a final score of 3 were assigned to the HC group; reactions with scores of 1 or 2 were assigned to the MC group; and reactions with a score of -1 were assigned to the NC group. Reaction scores of -1, 1, 2, and 3 also correspond to Not Detected, Low, Medium, and High expression levels expressed in Fig 2.

As an example, HADHB is expressed at low levels in cerebellum Purkinje cells; ACAA2 is not detected; and ACAA1 is expressed at high levels. The *r0634* gene-reaction rule mentioned above was then be replaced by “*MIN(1,MAX(-1,3))*”, which evaluates to 1. During the Purkinje cells reconstruction, this reaction was then placed in the MC group. Similar approaches have been used by previous studies to assign reaction confidence scores [30, 32, 37].

Aside from the four reaction groups, the CORDA algorithm also requires 5 parameters to operate, which are summarized in Table 4. To begin the algorithm, all HC reactions are moved into the tissue-specific reconstruction (RE), since these are sure to be included in the final model. Given the remaining three reaction groups, the CORDA algorithm proceeds in three steps:

1. MC and NC reactions associated with each RE reaction (which are the same as HC reactions at this point) are obtained using the dependency assessment *n* times. Here, each NC reaction is given a cost of *γ* and each MC reaction a cost of $\sqrt{\gamma }$, in order to favor the inclusion of MC over NC reactions. Any MC or NC reaction associated with any RE reaction, during any of the *n* dependency assessments, is then moved from the MC and NC groups to the RE group. These reaction associations are returned by the algorithm in order to assist in any subsequent manual curation.
2. NC reactions associated with each MC reaction are obtained using the dependency assessment *n* times. At this point, in order to maximize the inclusion of MC reactions and minimize the inclusion of NC reactions, we take a different approach than simply moving MC reactions and their associated NC reactions to RE. Instead, any NC reaction associated with *p* or more MC reactions is first moved from the NC group to the RE group. Subsequently, all remaining NC reactions are blocked (upper and lower bounds set to zero) and any MC reaction still able to carry flux in any direction, thus not depending on the blocked NC reactions, is moved from the MC to the RE group. This is done since, as described above, the blockage of NC reactions associated with a particular MC reaction does not necessarily remove that reaction’s ability to carry flux. All MC reactions not included in the reconstruction, as well as their associated NC reactions, are then returned by the algorithm to assist in any subsequent manual curation.
3. Lastly, all MC and NC reactions not yet added to the RE group are blocked. OT reactions associated with each RE reaction are then obtained, again using the dependency assessment *n* times. Any OT reaction associated with any RE reaction during any of the *n* dependency assessments is then moved from the OT to the RE group. This final RE group then defines the tissue-specific reconstruction.

It is worth noting that one of the main advantages of CORDA over pruning algorithms is the fact that it is independent of how reactions are ordered. This is due to the fact that reaction associations are calculated for each step, and at the end of each step a decision is made as to which reactions are added to the tissue reconstruction. This way, the order in which reaction dependencies are calculated does not affect the final tissue reconstruction.

The CORDA reconstructions used for comparison to previous methods were generated using *γ* = 10^5^, the highest cost value tested, *κ* = 10^-2^, the lowest noise value tested, *ϵ* = 1, a threshold similar to a previous study [32], *n* = 5, to allow for the inclusion of a larger number of OT reactions, and *p* = 2.

For a direct comparison to previous methods, the CORDA reconstructions used during the parameter sensitivity analysis, cross-validation, and comparison to previous methods were performed using the same data used for the mCADRE hepatocyte reconstruction. For the Monte-Carlo sampling analysis, a new reconstruction was generated using the most up-to-date data from the HPA. Both of these reconstructions are available in the supplemental material (S1 File). All calculations in this study were performed using the COBRA toolbox [129] and the Gurobi optimizer [128]. The MATLAB function file used for CORDA reconstructions is also available in the supplemental material (S2 File). Finally, an example of the CORDA algorithm, applied to small sample networks, is available in S2 Text.

### Parameter sensitivity analysis

While CORDA requires a number of different parameters, many of these values can be arbitrarily assigned. For instance, *γ* can be arbitrarily large, while *ϵ* and *κ* can be arbitrarily small. In order to demonstrate that the CORDA algorithm is robust to a wide range of parameters, we performed 108 hepatocyte specific reconstructions varying all parameters but *p* (which was set to be equal to two) to a wide range of values. A separate sensitivity analysis of *p* was performed and is included in S1 Text. The parameter *p* can be set in order to define a more or less flexible MC and NC core, and can be set to the user’s discretion.

These 108 reconstructions were based on the generalized human reconstruction Recon1 [5], using the same set of protein expression data (total of 560) and 32 of the metabolic tests used in the mCADRE hepatocyte specific reconstruction [30]. The data used in this step, as well as the metabolic tests and calculated reaction groups, are available in the supplemental information (S1 Table). Metabolic tests were included as single reactions in the reconstruction in order to assure the model was able to produce certain metabolites. Each metabolic test was added to the model when being tested then immediately removed, so that no two tests were present in the model at the same time, and no metabolic test reaction was included when other reactions were being assessed. Details of this analysis are available in S1 Text.

### Metabolic tests analysis

During the metabolic tasks validation analysis, the exchange rate of the *basal inputs* carbon dioxide (co2[e]), water (h2o[e]), protons (h[e]), oxygen (o2[e]), phosphate (pi[e]), hydrogen peroxide (h2o2[e]), superoxide anion (o2s[e]), bicarbonate (hco3[e]) and carbon monoxide (co[e]) were unconstrained. All other uptake reactions were blocked unless otherwise specified.

For each of the 20 amino-acid recycling tests, the uptake rate of the given amino acid and glucose were set to an arbitrary value, so that the amino-acid being tested was the only source or nitrogen. Next, the production of urea was set to a strictly positive value, and FBA was performed while optimizing the production of urea. The same test was also performed for ammonium. For each of the 21 glucogenic tests, the uptake rate of the given metabolite was set to an arbitrary value, and the production of glucose was optimized. For both the amino-acid and glucogenic tests, if the model returned a feasible flux distribution the test was considered passed, otherwise it was considered failed. If the exchange reaction of the given metabolite was not present in the model, the result was considered inconsistent. The generalized Recon1 reconstruction failed two of the glucogenic tests, so the results of the remaining 19 tests are reported in the main text.

For the eight nucleotide production tests, a sink consuming the given nucleotide was added to the cytosolic compartment. The model was allowed to uptake glucose and ammonium (as a source of nitrogen), and the flux through the sink was optimized. If the model was able to produce the given nucleotide, the test was considered passed.

### Generation of healthy and cancer tissue models

Following the validation of the CORDA algorithm, we generated a library of 76 healthy and 20 cancer tissue-specific reconstructions using the generalized human reconstruction Recon2 [9] and the most recent proteomics data from the HPA [44, 45]. All reactions used to generate the tissue-specific models are available in S1 Table, and tissue-specific models are available in SBML and MATLAB format at [130]. The healthy tissue models were calculated using the same classification as described in the algorithm description section, since data for each protein was categorized as not detected, low, medium or highly expressed in each cell type. For cancer models, the same classification was available for any number of samples for each protein in each cancer type. In this case, values of -1, 1, 2 and 3 were assigned to each sample according to not detected, low, medium or high expression levels respectively, and these values were averaged for a final protein score in that particular cancer type. These protein values were then used in the gene-reaction boolean association as described in the algorithm description for a final reaction score. Reactions with a score equal to or greater than 2.5 were assigned to the HC group, less than 2.5 but greater than 1 to the MC group, and less than or equal to -0.5 to the NC group.

For instance, in renal cancer samples, protein *HADHB* has been analyzed in 12 different samples in the HPA, and was found to be expressed in high levels in 2 of them, medium levels in 8, and in low levels in 2. The protein score associated with *HADHB* in renal cancer is then calculated as $\frac{\left(2\cdot 3)+(8\cdot 2)+(2\cdot 1\right)}{12}=2$. Similarly, *ACAA1* expression was calculated as medium in 5 samples, low in two samples, and not detected in four samples of renal cancer, yielding a score of $\frac{\left(5\cdot 2)+(2\cdot 1)+(4\cdot (-1)\right)}{11}=0.73$. Finally, *ACAA2* is present in high levels in one sample, medium level in 5 samples, low levels in one sample and not detected in 3 samples of renal cancer, giving this protein a score of $\frac{\left(1\cdot 3)+(5\cdot 2)+(1\cdot 1)+(3\cdot (-1)\right)}{10}=1.1$. With that, the score for *r0634* is calculated as “*MIN(2,MAX(0.73,1.1))*”, which is 1.1, putting this reaction in the MC group during the renal cancer reconstruction. Data and reaction distributions used during these calculations can be found in S1 Text.

### Clustering of healthy and cancer tissue models

Healthy and cancer specific models were clustered according to reactions present in each model. For that, 4,205 reactions present in at least one, but not all models were obtained. A binary vector was then calculated for each model indicating whether reactions were present (1) or not present (-1). These vectors were then clustered using hierarchical clustering with Hamming distance as the similarity metric, and average linkage. Leaf orders were also calculated in order to maximize the similarity between neighbors in the hierarchical binary cluster tree dendrogram. These results are summarized in Fig 4.

Next, in order to divide the clusters according to subsystem expression, a total of 4,751 reactions present in any of the models was obtained. These reactions were then divided by subsystem according to their classification in the Recon2 reconstruction. For each of the clusters of models calculated in the previous step, the average number of reactions from each subsystem included in the cluster’s models was then calculated. Finally, this number was divided by the total number of reactions in that subsystem which were included in any of the models for a final score between zero and one. These values were then clustered using hierarchical clustering with Euclidean distance as the similarity metric, and average linkage. Leaf orders were again organized to maximize similarity between neighbors to yield Fig 5.

### Flux Balance Analysis

Perhaps the most widely used method to analyze GEMs is Flux Balance Analysis (FBA) [42]. FBA predicts a flux distribution through the metabolic network which optimizes (maximizes or minimizes) a given *objective function*, defined as a single reaction or group of reactions in the network. This flux distribution is subject to upper- and lower-bound constraints, which include exchange reactions, and a steady state assumption for all model metabolites, so that no metabolite has a net production or consumption rate.

The mathematical formulation of GEMs are defined at the core by a stoichiometric matrix *S*, where each row defines a metabolite, each column defines a reactions, and each entry the stoichiometric coefficient of that metabolite in that particular reaction. Vectors defining lower (*lb*) and upper (*ub*) bounds for each reaction, as well as an objective vector (*c*) of the same length, are also defined. Given this model, FBA finds a flux vector *v* through all reactions in the GEM such that:

$$\begin{array}{c} S\cdot v=0\\ lb_{i}\leq v_{i}\leq ub_{i}\\ optimize:v\cdot c^{T} \end{array}$$

During the dependency assessment described here, the stoichiometric matrix *S* is altered to reflect the changes described above. Given a reaction *j* being tested, a group of undesirable reactions *Y*, and a matrix *S* of size *m* by *n*, let $\ddot{\kappa }$ denote a random number drawn uniformly between 0 and *κ*. The GEM is modified in the following ways:

1. Split all reversible reactions: *if* (*lb*_*i*_ ≤ 0&;*i* ≠ *j*)→*S*(:, *n*+1) = −*S*(:, *i*), *lb*_*n*+1_ = 0, *ub*_*n*+1_ = −*lb*_*i*_ and *lb*_*i*_ = 0, where *S*(:, *n*+1) denotes the addition of a column (reaction) to *S*.
2. Add cost to reactions: $if\;\left(i\in Y\right)\to \;S\left(m+1,i\right)=\gamma +\ddot{\kappa };else\to \;S\left(m+1,i\right)=\ddot{\kappa }$, where *S*(*m*+1, :) is the row denoting the cost pseudo-metabolite.
3. Add cost consuming reaction: *S*(:, *n*+1) = [0 0 0 … 0 − 1]
4. Set bound of reaction *j* to desired value: *ifϵ* > 0 → *lb*_*j*_ = *ϵ*; *else* → *ub*_*j*_ = *ϵ*
5. Set objective to cost consumption: *c* = [0 0 0…0 1]

With these constraints in place, FBA is performed as described above while minimizing the objective function. For each reaction in the reconstruction, if *i* ∈ *Y* and *v*_*i*_ ≠ 0, the reaction *i* is deemed associated with *j*.

### Monte-Carlo sampling

Monte-Carlo sampling was performed in a manner similarly to Bordbar et. al.[25] and Lewis et. al.[49]. This sampling method is a slight variation of the Artificially Centered Hit and Run (ACHR) algorithm developed by Kaufman and Smith [131]. In this algorithm, warmup points are initially generated at random corners of the solution space by solving an LP problem with objective vectors containing randomly generated ones and negative ones. The center point between all points is then computed. Next, for each point sampled, a random direction is selected as the difference between a randomly selected point and the center point. By selecting the direction this way, the direction is biased in the longer direction of the solution space, speeding up the rate of mixing while maintaining uniformity. After a direction is chosen, the limit of how far the current point can travel in that direction is calculated, and a new point is randomly chosen along that line. After several iterations, the set of generated points will be well mixed and approach a uniform sampling of the solution space.

The termination condition imposed on the ACHR algorithm here is the same imposed by Bordbar et. al.[25] and Lewis et. al.[49], introducing the concept of mixed fraction. For that, a partition is created over the set of points by drawing a line at the median value, with half the points on either side of the partition. The mixed fraction is the number of points that cross this line during mixing. Initially, the mixed fraction is one as all the points are on their original side of the line. As the sample solutions are mixed, the probability of each point crossing the median line approaches 0.5 asymptotically. The sampled points were initially mixed using the warmup points created as described above until the mixed fraction reached a particular threshold. Following that, the samples were mixed two more times, using the previous iteration’s final points as warmup points, until the same mixed fraction was reached. For the comparison between CORDA and other tissue-specific algorithms, a mixed fraction threshold of 0.52 was chosen as the termination condition. For the cancer and healthy tissue-specific models, a mixed fraction threshold of 0.6 was chosen to make the 96 sampling experiments computationally feasible.

Due to the heterogeneity between tissue-specific models, sampled flux values were evaluated between all cancer and healthy tissue models separately. That is, all sampled flux values for the given reaction were obtained from all cancer models that contain that reaction, and compared to all sampled values from healthy tissue models that contain the reaction. Results of this analysis are presented in Fig 6. In some cases, two or more reactions were combined: MTHFD2* combines reactions MTHFD2 and MTHFD2m, GHMT2r* combines reactions GHMT2r and GHMT2rm, and SPODM* combines reactions SPODM, SPODMe, SPODMm, SPODMn and SPODMx. These are the same reactions taking place in different cellular compartments. For these, flux values from each of these groups of reactions were added within each sampled flux distribution when plotting Fig 6.

## Supporting Information

S1 Text Supplementary Text 1. Supplementary text includes details of parameter sensitivity analysis, hypergeometric p-value calculations, parameter *p* sensitivity analysis, comparison between CORDA, MBA and mCADRE, healthy and cancer tissue reconstructions process, and Monte-Carlo sampling analysis.
(PDF)

S2 Text Supplementary Text 2. Example of application of CORDA to small sample networks.
(PDF)

S1 Table Supplementary Tables. Table contains the proteomics data and reaction groups used to calculate the Hepatocyte reconstructions, 32 metabolic tests used throughout the manuscript, reactions used to calculate tissue-specific models, reactions uniquely included in healthy and cancer reconstructions, and essential metabolite results.
(XLSX)

S1 File Liver CORDA models. Reference hepatocyte specific reconstructions calculated using CORDA and Recon1. Two reconstructions are available, one using the same protein dataset used in the mCADRE reconstruction, and one using the most recent protein data in the HPA (S1 Table). The parameters used in this reconstruction are *γ* = 10^5^, *κ* = 10^-2^, *ϵ* = 1, *p* = 2, and *n* = 5.
(ZIP)

S2 File CORDA file. MATLAB function used for CORDA reconstructions.
(ZIP)
