# Supplementary material for: Reconstruction of Tissue-Specific Metabolic Networks Using CORDA
Source: PLoS Comput Biol. 2016 Mar 4;12(3):e1004808. doi: 10.1371/journal.pcbi.1004808 (PMC4778931; doi:10.1371/journal.pcbi.1004808)
Supplement: S1 Text — Supplementary text includes details of parameter sensitivity analysis, hypergeometric p-value calculations, parameter p sensitivity analysis, comparison between CORDA, MBA and mCADRE, healthy and cancer tissue reconstructions process, and Monte-Carlo sampling analysis. (PDF) [file pcbi.1004808.s001.pdf]

Parameter Range Analysis

Parameters Tested

To test the robustness of CORDA to parameter values, we reproduced 108 hepatocyte specific tissue reconstructions using a wide array of parameters. First, we constrained the flux value of the reaction being tested by either specific values or percentage from maxima. Under six different conditions, ε was constrained to be 0.1, 1 or 5, or 1, 5 or 10% of the reaction's maximum flux range. We also allowed γ to be 10^3, 10^4 or 10^5, $\varkappa$ to be 10^{-1} or 10^{-2} and n to be 1, 5 or 10. We found that higher orders of magnitude of γ would lead to a scaling error. p was not varied in this section, but a separate sensitivity analysis of this parameter is provided below. By combining the six flux values, two noise values, three cost values and three sampling time values; we obtained a total of (6*2*3*3) = 108 liver models.

Results

Our analysis defined 350 HC reactions, which, as expected, were included in all the models; 344 MC reactions, 338 (98.3%) of which were included in all the models; and 79 NC reactions, 16 (20.25%) of which were included in all 108 models (S1 Table). Next we analyze each MC reaction excluded and each NC reaction included in different models according to their protein expression level in the Human Protein Atlas (HPA)[1,2] and the mCADRE data. Proteins in the HPA are categorized as *supportive* or *uncertain*, according to level of confidence vested in the results present in the database.

MC Reactions

In Recon1, there were 350 HC reactions able to carry flux. Unsurprisingly, all of those reactions were included in all the models calculated (as this was part of the algorithm). There were also 344 MC reactions, 338 (98.3%) of which were included in all the models. Four MC reactions were included in none of the models. These were:

- **AMANK** – the gene-reaction rule specified for AMANK in Recon1 is defined as 10020 (GNE) or 55577 (NAGK). GNE is defines as medium expression in hepatocytes. NAGK, on the other hand, is defined as low expression in the mCADRE data, but has since been changed to "not present" in the liver tissue. In the Human Protein Atlas, the evidence for GNE is uncertain, while NAGK is supportive. AMANK depends on the reaction ACNAM9PL. ACNAM9PL is defined by the gene 54187 (NANS), which is not identified in the liver tissue. NANS's evidence is uncertain according to the HPA.
- **GND** – GND is dependent on the gene 5226 (PGD). PGD is expressed in low levels in the liver tissue according to the HPA. PGD is dependent on G6PDH2r, which depends on the gene 2539 (G6PD). This gene is not detected in hepatocytes according to the HPA. Evidence for G6PD is supportive in the HPA, while PGD is not.
- **PGDI** – PGDI is dependent on 5730 (PTGDS) or 27306 (HPGDS). 27306 is defined as lowly expressed in the liver according to the mCADRE data, while 5730 is not present. Currently, however, HPGDS is defined as "not present" in the liver tissue, although PTGDS is lowly expressed. Evidence for both is categorized as uncertain. PGDI depends on PGS. PGS is defined by 5743 (PTGS2) or 5742 (PTGS1). Both of these are not detected by supportive evidence in hepatocytes according to the HPA.
- **PI3P4K** - This reaction depends on many genes. These are:
    - 55361 (PI4K2A) - not in the mCADRE data and with uncertain low expression in the HPA.
    - 5297 (PI4KA) - not in the mCADRE data and with uncertain low expression in the HPA.
    - 8394 (PIP5K1A) - uncertain medium expression in the HPA.
    - 23396 (PIP5K1C) - not in the mCADRE data and with uncertain low expression in the HPA.
    - 8395 (PIP5K1B) - not in the mCADRE data and with uncertain low expression in the HPA.

- 5298 (PI4KB) - uncertain medium expression in the HPA.

PI3P4K is dependent on PI345P3P, however, which is dependent on the gene 5728 (PTEN). PTEN is not detected in hepatocytes, also with uncertain evidence, according to the HPA.

The remaining two MC reactions were included in some of the models. These were:

- **ACONTm** – ACONTm is included in only two reconstructions. This reaction is dependent on the gene 50 (ACO2). ACO2 is expressed at high or medium levels in 66 of the 74 normal tissues analyzed by the HPA, but it is expressed at a low level in hepatocytes according to supportive data. ACONTm also depends on either ICDHxm or ICDHyrm, both of which are not present in hepatocytes according to supportive data in the HPA (see below).
- **TRDR** – Present in 69 models. This reaction depends on the gene 7296 (TXNRD1), expressed at medium levels in hepatoctytes according to supportive data. TRDR is dependent on RNDR3. RNDR3 depends on 6240 (RRM1) and 6241 (RRM2), both of which are not expressed in hepatocytes according to suggestive, but uncertain HPA data.

Three out of the four reactions excluded from all models are supported by uncertain experimental data, while they depend on reactions that are not present according to supportive evidence. The exclusion of these reactions exemplifies CORDA's ability to remove reactions with little, inconclusive experimental evidence, based on stronger opposing evidence. The presence or non-presence of these reactions should be considered as hypotheses generated by our tissue reconstructions. That is, whether these reactions do take place in hepatocytes should be tested and the model network topology should be revisited to assess the reaction dependency determined here. This analysis also demonstrates the values of the set of reactions returned by our model in assisting manual curation, given that all these reactions and their associations were returned by CORDA when the reactions were not included.

NC Reaction

There are a total of 79 NC reactions able to carry flux in the Recon1 reconstruction. Out of these reactions, 16 (20.25%) were present in all tissue reconstructions. These are:

- **ACGAM2E** – Dependent on 5937 (RENBP), not detected in hepatocytes according to uncertain data.
- **AMETt2m** – Dependent on gene 115286 (SLC25A26), not detected in hepatocytes based on supportive evidence. SLC25A26 was present in medium or high quantities in 41 of the 79 healthy tissues analyzed.
- **BAAT1x**, **BAAT3x**, **BAAT4x** – Dependent on 570 (BAAT), defined as not detected in the mCADRE data, but now specified as highly expressed in hepatocytes according to supportive data.
- **CSNAT2m**, **CSNAT3x**, **CSNATm** and **CSNATp** – Defined by 1384 (CRAT) not detected based on supportive data.
- **DHCR72r** – Defined by 1717 (DHCR7), specified as not detected in the mCADRE data, but now specified as lowly expressed in hepatocytes according to supportive data.
- **MTHFD2m** – Dependent on 10797 (MTHFD2) not detected based on supportive evidence.
- **NDPK10n**, **NDPK4n** and **NDPK9n** – 4831 (NME2), not present in the mCADRE dataset but expressed in low levels in hepatocytes according to the HPA, and 4830 (NME1), defined as not detected in the mCADRE data, but now defined as lowly expressed in hepatocytes. Both are now defined by supportive data.
- **P4507A1r** – Dependent on 1581 (CYP7A1), defined as not expressed in hepatocytes according to the mCADRE data, but currently not specified in the HPA at the protein level. RNA expression can be observed only in liver tissue, however.
- **TAURt4(2)r** – Dependent on gene 6533 (SLC6A6), not detected in hepatocytes according to supportive evidence.

Therefore, out of the 16 NC reactions corresponding to 9 gene-reaction association rules included in all models, as defined by the mCADRE data, 7 of these reactions, corresponding to three gene-reaction rules, have changed to

being supported by the HPA at the protein level. Additionally, one reaction has since changed from negative protein evidence to non-existing evidence, but is supported at the gene expression level. The fact that a high percentage of the NC reactions added to all models have since changed to having protein data associated with them demonstrates the power of CORDA to include needed reactions as well as the importance of a flexible core set of reactions.

An additional 12 NC reactions were present in some of the models, most in two or less of the 108 models. These 12 reactions are:

- **RNDR1** - Present in 69 models. Dependent on 6240 (RRM1) or 6241 (RRM2), both not found in hepatocytes according to uncertain data.
- **NADH2_u10m** - Present in 16 models and dependent on a large number of genes.
- **PIt9** - Present in 10 models. Dependent on 142680 (SLC34A3), not found in hepatocytes. Supportive evidence.
- **ICDHyrm** - Present in two models. Dependent on 3418 (IDH2), not found in hepatocytes according to supportive data.
- **NDPK1n,NDPK2n**, **NDPK3n**, present in two models, and **NDPK7n**, present in one model - Dependent on 4830 (NME1) and 4831 (NME2). NME1 is indicated as not present in the mCADRE data. As stated above, both NME1 and NME2 are indicated as present in low quantities in the liver according to supportive HPA data, however.
- **ADPRIBt** and **NADNe** - Present in one model. Dependent on 952 (CD38), not present in the liver according to supportive data.
- **ICDHxm -** Present in one model. Dependent on 3419 (IDH3A), not present in hepatocytes, 3420 (IDH3B), low expression in hepatocytes, and 3421 (IDH3G), not present in hepatocytes. All according to supportive data.
- **PCHOLP_hs -** Present in one model. Dependent on 5338 (PLD2), not detected in hepatocytes based on supportive evidence.

Comparison Between Models

We next compare the composition of each of the 108 models according to the parameters used to calculate them. **STable1** presents the number of varying reactions present in each model, that is, how many of the two MC and 12 NC reactions that appeared in some but not all models are present in each reconstruction. Two models contained

		Constraint by value						Constraint by percentage					
		0.1		1		5		1		5		10	
		Noise Value						Noise Value					
Magnitude of Cost	Numer of times Simulated	1.00E-01	1.00E-02	1.00E-01	1.00E-02	1.00E-01	1.00E-02	1.00E-01	1.00E-02	1.00E-01	1.00E-02	1.00E-01	1.00E-02
1,000	1	1/1	1/1	0/0	1/1	1/1	1/1	0/0	0/0	0/0	0/0	0/0	1/1
	5	0/0	1/3	1/2	1/1	1/1	1/1	1/3	1/2	1/1	1/2	1/1	1/1
	10	1/2	1/1	1/1	1/1	1/1	1/2	1/1	1/3	1/2	1/1	1/1	1/1
10,000	1	0/0	0/0	0/0	0/0	0/0	0/0	0/0	0/0	0/1	0/0	1/1	0/0
	5	1/1	1/1	1/1	0/0	1/2	1/1	0/0	1/2	1/1	1/2	1/1	0/0
	10	0/1	1/1	1/1	0/0	1/1	0/1	1/2	1/2	1/2	2/3	1/1	1/2
100,000	1	0/0	0/0	0/0	0/0	0/0	0/0	0/0	0/1	1/1	1/1	0/0	0/0
	5	1/1	1/1	0/1	1/1	1/1	1/1	0/0	0/2	1/1	1/2	0/0	1/1
	10	1/1	1/1	2/3	1/1	0/2	1/1	1/1	0/1	1/2	1/3	1/2	1/2

Reconstructions Containing *ACONTm*. All other reconstructions containing an alternative MC reaction contain TRDR.
Reconstructions containing NDPK1n, NDPK2n, NDPK3n and/or NDPK7n
Reconstructions containing ICDHxm, ICDHyrm, PCHOLP_hs, NADNe and/or ADPRIBt
Reconstruction containing NADH2_u10m and/or PIt9

STable1: Numbers are defined as MC reactions added to the model / NC reactions added to the model. These numbers refer only to reactions added to some of the models, that is, reactions that are included in at least one, but not all models. We can see that the difference between models varies by very few reactions.

both varying MC reactions, leaving 106 models with one or zero of these MC reactions. Out of the 12 NC reactions, 6 of the models contained 3 of those reactions, leaving 102 models with two or fewer varying NC reactions.

We can also see that reconstructions built using fluxes constrained by the given percentages are more prone to include the NADH2_u10m and PIt9 reactions. Considering that many of the fluxes have an upper bound of 1,000, constraining this value to 5 or 10%, for example, would lead to a relatively high flux through the reaction being tested.

OT Reactions

We next analyzed the presence of OT reactions in each model. In total, there are 1,696 OT reactions able to carry flux in Recon1. Of these, 1,216 were present in at least one reconstruction. Of these 1,216 reactions, 1,056 (86.84%) appeared in all models. **STable2** presents how many of the remaining 160 reactions appeared in each of the reconstructions. We can see that, as expected, performing the reconstruction using a higher value of *n* yields a higher number of OT reactions included, or alternative pathways. When considering only reactions simulated multiple times, 1,102 (90.62%) of the 1,216 reactions were included in all models.

		Constraint by value						Constraint by percentage					
		0.1		1		5		1		5		10	
		Noise Value						Noise Value					
Magnitude of Cost	Numer of times Simulated	1.00E-01	1.00E-02	1.00E-01	1.00E-02	1.00E-01	1.00E-02	1.00E-01	1.00E-02	1.00E-01	1.00E-02	1.00E-01	1.00E-02
1,000	1	73	65	70	74	68	75	67	76	69	66	63	77
	5	105	105	113	107	108	105	113	101	104	107	106	111
	10	113	116	112	120	113	113	113	113	114	115	115	113
10,000	1	68	68	68	66	57	70	71	68	66	71	71	62
	5	103	105	110	101	105	110	102	108	101	113	107	111
	10	107	115	111	113	111	110	108	120	114	100	113	120
100,000	1	67	70	65	69	61	64	69	64	72	66	69	67
	5	109	104	102	107	103	108	102	110	108	98	106	111
	10	111	111	94	111	104	113	115	110	119	120	117	112

STable2: Numbers refer to OT reactions added to each model. We can see that using *n*=1 leads to the inclusion of fewer OT reactions, while using higher values leads to the inclusion of significantly more alternative pathways. The numbers presented refer only to reactions added to some of the models; that is, reactions that are included in at least one, but not all of them.

The number of OT reactions included in the reconstruction can then be defined to a certain extent by the parameter *n*. If we wish to perform a more parsimonious reconstruction, with fewer reactions, we can perform the reconstruction using *n*=1, whereas if we would like to include more alternative pathways, a higher value of *n* can be used. The 36 models generated by assessing each reaction dependency a single time contained on average 1,828.7 reactions, while five simulations returned 1,868.3 and ten returned 1,874.9.

It is worth noting that even by simulating each model a single time, the reconstruction returned by CORDA is not as concise as those returned by other methods such as MBA and mCADRE. That is due to the fact that here, the shortest, or at least most cost effective pathway is included for each reaction already in the reconstruction, while in the aforementioned methods, longer pathways may be present that satisfy a larger number of pathways.

Metabolic Task Results for All Models

We next performed the same set of tests performed during the comparison between CORDA, mCADRE and MBA, on the 108 CORDA models produced here. We find that all models presented the same results for all amino-acid recycling tests and glucogenic tests. The only difference between all 108 CORDA reconstructions performed here is in the Nucleotide production tests. We found that 69 of the 108 (63.9%) models were able to produce the nucleotides dATP, dCTP and dGTP, while the remaining 39 models were not. This discrepancy was traced back to the inclusion of the reaction TRDR in the 69 successful models. This is a Medium confidence reaction that is dependent on the negatively expressed reaction RNDR1.

Overall, however, the models returned the same results on 47 of the 50 metabolic tests (94%), demonstrating that model functionality is robust to noise and algorithm parameters. This discrepancy, however, highlights the importance of (1) including metabolic test results in the reconstruction process, which can be done in the CORDA algorithm, and of (2) returning a set of dependent reactions following the model building process, which is also done by CORDA.

Parameter Range Analysis Summary

In summary, CORDA was able to include 98.3% of the reactions in Recon1 with medium or low level of confidence, while including only 20.25% of the reactions determined as not present. Furthermore, upon closer inspection, half of those NC reactions added have since changed to have supporting evidence, demonstrating a good ability of CORDA to include necessary reactions with contradicting evidence. Finally, we demonstrated that the number of OT reactions, or generally supporting reactions, can be tailored through model parameters. Throughout the manuscript, we use the model calculated using $\varepsilon=1$, $\gamma=10^5$, $\varkappa=10^{-2}$ and $n=5$.

Parameter "*p*" sensitivity analysis

We have also tested the sensitivity of the algorithm on the parameter *p*, which defines the flexibility of the reactions core during the second step of the CORDA algorithm. For this analysis, we used the parameters $\varepsilon=1$, $\gamma=10^5$, $\varkappa=10^{-2}$ and $n=5$ to first perform step one of the CORDA algorithm. Next, the dependency assessment method was used to calculate the dependency of each MC reaction on the NC reactions. Out of the 96 NC reactions at this point, 84 were found to be associated with no MC reaction, 8 were associated with one MC reaction, and 4 were associated with two MC reactions.

Using these conditions as our starting point, we proceeded with the algorithm using three different values of *p*: 1, 2 and 3, generating three hepatocyte models. A value of zero would lead to the inclusion of 84 NC reactions associated with no MC reactions, while a value of 4 would have the same effect as 3, leading to the exclusion of all remaining NC reactions at this point. The reconstruction using *p*=3 led to the inclusion of 13 NC reactions, all of which were added at step 1 of the CORDA algorithm. As expected, using *p*=2 led to the inclusion of an additional four NC reactions (17), and using *p*=1 led to the inclusion of an additional 12 NC reactions (25). Surprisingly, however, different values of *p* had little effect on number of MC reactions included and overall model size (**SFig1**). Using *p*=2 led to the inclusion of 7 more MC reactions, and using *p*=1 led to the inclusion of 12 more MC reactions over *p*=3. For model size, using *p*=2 led to the inclusion of 30 more reactions, and using *p*=1 led to the inclusion of 34 more reactions over *p*=3

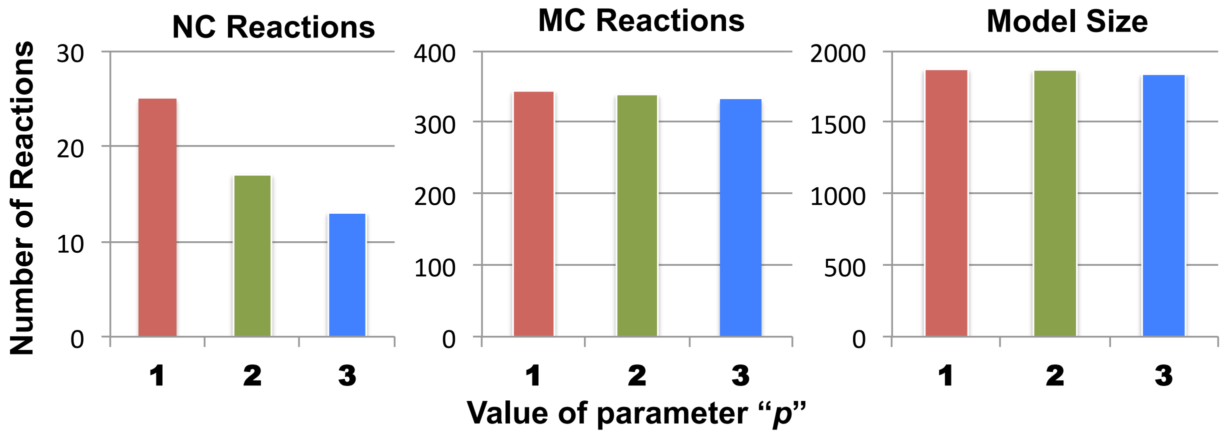

SFig1: For *p*=1 the model has 1871 reactions, 344 MC and 25 NC. For *p*=2 the model has 1867 reactions, 339 MC and 17 NC. For *p*=3 the model has 1837 reactions, 332 MC and 13 NC.

We next performed the same set of functionality analysis described in the main text in each of these three models. We found that the reconstructions performed using *p*=1 and 2 had the same results as the CORDA model presented in Table2 of the main text, and further described in the next sections. The reconstruction using *p*=3 led to the exclusion of the reaction *TRDR*, causing this model to fail three of the nucleotide production tests.

Overall, we have shown that in this example the parameter *p* has a small range of variability, that leads to small differences in number of MC reactions included in the final model and overall model size. It is worth noting that results might be different for other tissue-specific reconstructions. Depending on the data available and desired reconstruction (more or less parsimonious), CORDA users can specify the value of parameter *p*. This can be done by analyzing the dependency assessments returned by the CORDA algorithm, evaluating how MC reactions are dependent on the NC group at the second step of the reconstruction process. Throughout the manuscript, we use the middle value of *p* defined here, *p*=2.

Calculation of cross-validation statistical significance

As described in the main text, 100 hepatocyte specific metabolic reconstructions were performed using randomly sampled subsets of the data used in the reconstruction of the previous 108 models. Here, randomly selected 280 of the 350 HC reactions, 275 of the 344 MC reactions, and 63 of the 79 NC reactions (80% of each group) were used in each of the 100 new models. All reactions not sampled from each of these groups were ultimately included in the OT reactions group before the reconstruction process.

Hypergeometric p-values were calculated for each tissue-reconstruction based on the number of reactions left out in each group, but ultimately included in the reconstruction, using the MATLAB function *hygecdf*. For instance, assuming we start the reconstruction process with N OT reactions, which include K HC reactions (the ones randomly left out of the reconstruction process), and finish with n OT reactions in the final reconstruction, of which k are from the K HC reactions. The hypergeometric p-value is calculated as:

$$1 - hygecdf(k-1, N, K, n)$$

Since *hygecdf(k-1,N,K,n)* gives the probability of randomly including *k-1* or fewer HC reactions in the final model, the expression above gives the probability of including k or more. The mean hypergeometric p-values calculated were 0.03 for HC reactions, 0.16 for MC reactions and 0.71 for NC reactions.
Once all 100 p-values were calculated for each reaction group (one for each of the 100 models generated here), these values were combined using Fisher's method. First, the test statistic t was calculated as:

$$t = -2\sum_{i=1}^{n} ln(p_i)$$

Using the MATLAB code t=-2*sum(log(p)). In order to calculate the combined p-value, we then used the expression:

$$p = 1 - F_{\chi^2}(t; 2n)$$

Where $F_{\chi^2}(x;k)$ is the cumulative distribution function for a χ^2 distribution with k degrees of freedom. This was done in MATLAB as 1-cdf('Chisquare',t,200). If the value returned was zero, the test was considered statistically significant (which was the case for the HC and MC groups), if the value returned was 1, it was considered not significant (which was the case for the NC group).

The figure below plots all calculate p-values for all 100 reconstructions calculated here:

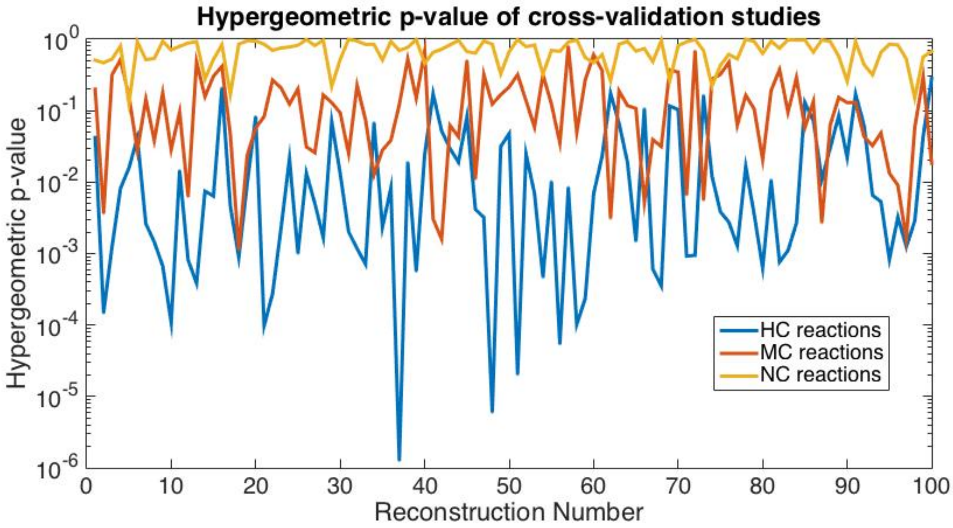

SFig2: p-values calculated for all 100 cross-validation reconstructions. HC reactions are most significantly included, followed by MC reactions. NP reactions are not preferentially included.

Distribution of Unique Genes and Metabolites

The following Venn diagrams plot the overlap between unique genes and unique metabolites between the three hepatocyte-specific reconstructions (MBA, mCADRE and CORDA1). A more significant overlap is seen between these categories than between reactions, with at least 77% of unique genes in each model present in all models, and at least 84% of unique metabolites in each model present in all models. Furthermore, no significant bias is observed between models; that is, no two models seem more alike than the other.

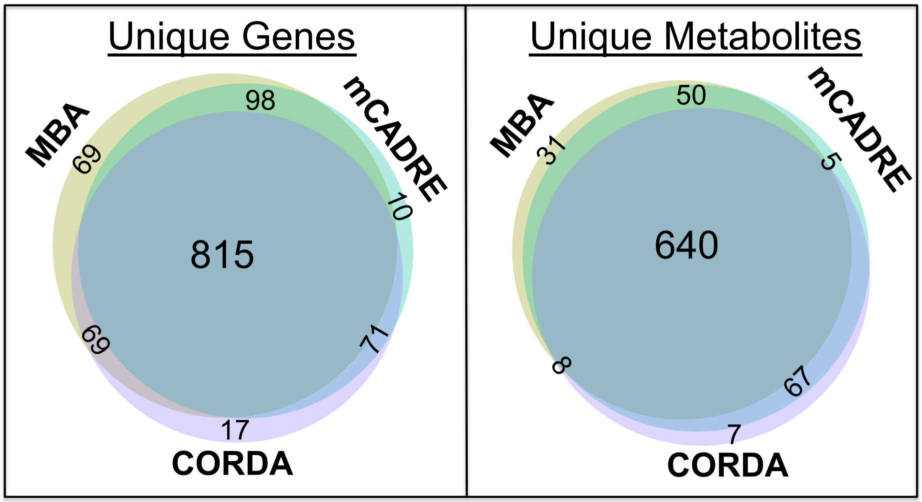

SFig3: Venn diagrams demonstrating the overlap between unique genes (left) and unique metabolites (right) included in each of the three models.

Composition of Models

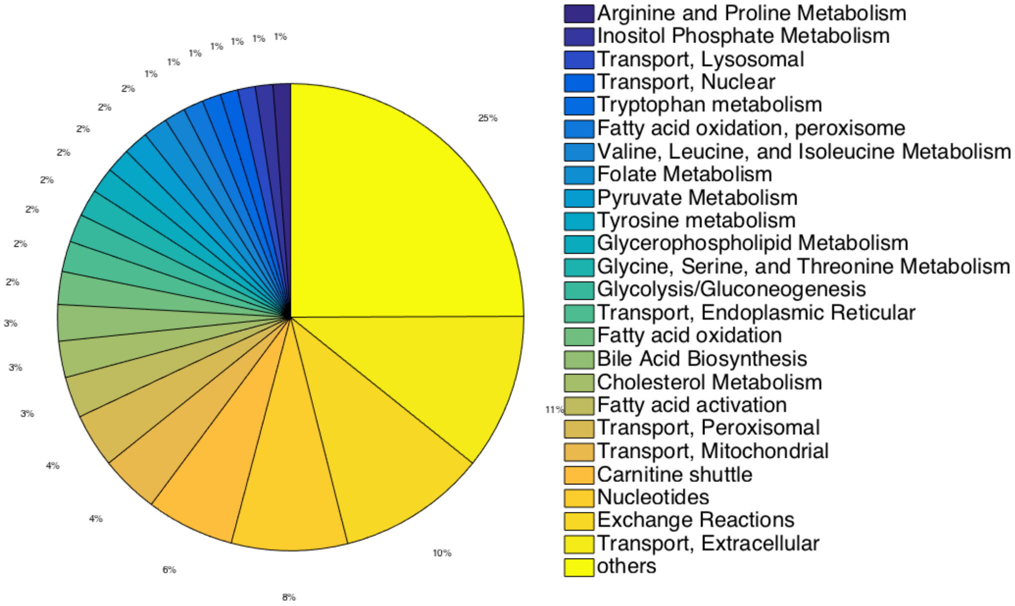

SFig4: Subsystem of reactions included in all three hepatocyte-specific models.

Next we plotted Venn diagrams of the subsystems of each reaction included in each model. **SFig4** shows the subsystems of reactions included in all models, **SFig5** shows the subsystem of reactions included in a single model, and **SFig6** shows the subsystems of reactions included in two of the models only. We can see a wide distribution of reaction subsystems included in all models (**SFig4**), with a large amount of exchange and transport reactions overlap. CORDA1 has a large amount of transport and exchange reactions unique to the reconstruction, as well as R group synthesis reactions (**SFig5**). MBA contains a large amount of unique amino-acid and glycerophospholipid metabolism reactions, and mCADRE contains a few unique steroid metabolism reactions (**SFig5**). Reactions lacking in MBA are widely distributed between subsystems, while mCADRE lacks a large number of transport and exchange reactions, and CORDA lacks nucleotide metabolism reactions when compared to other models (**SFig6**).

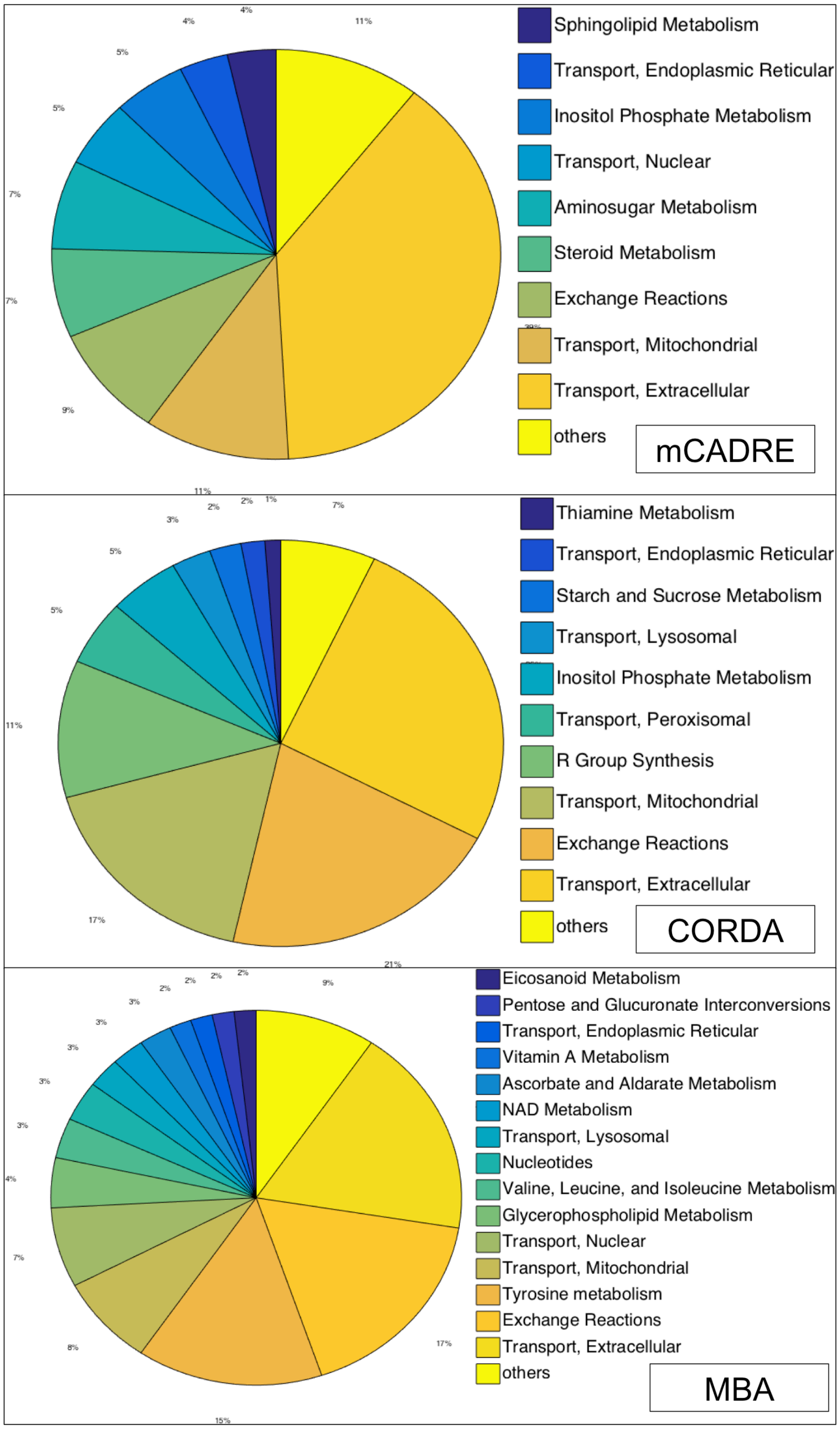

SFig5: Subsystem of reactions present in only one of the models.

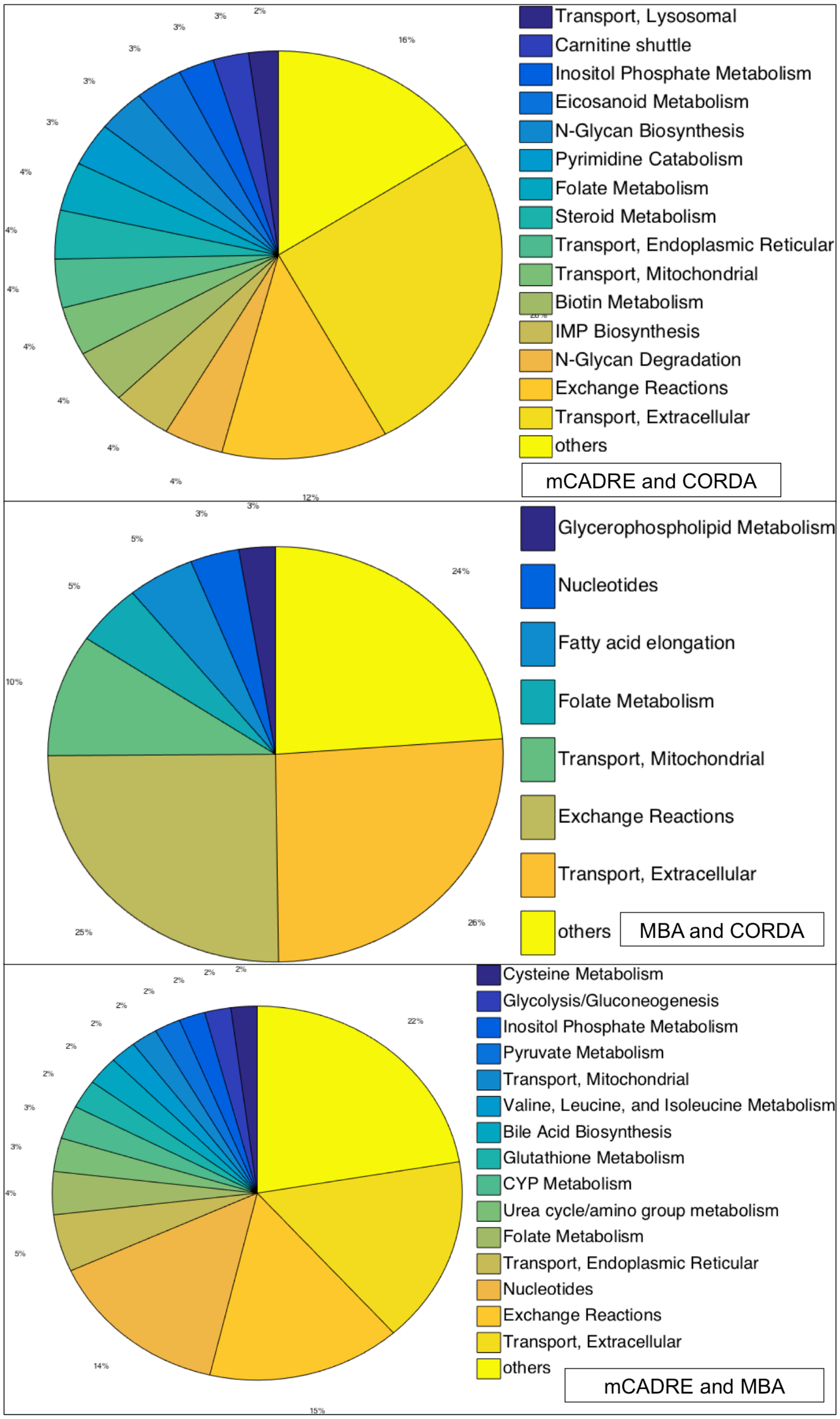

SFig6: Diagram of subsystems of reactions included in only two of the models.

Metabolic Tests

We submitted each of the three models to a series of metabolic tests. For each of these tests, the model was allowed to uptake a series of currency metabolites, if such exchange reaction was in the model, which we will refer to as *basal inputs*. These include carbon dioxide (*co2[e]*), water (*h2o[e]*), protons (*h[e]*), oxygen (*o2[e]*), phosphate (*pi[e]*), hydrogen peroxide (*h2o2[e]*), superoxide anion (*o2s[e]*), bicarbonate (*hco3[e]*) and carbon monoxide (*co[e]*). All other uptake reactions were set to zero unless otherwise specified. While in the main manuscript we compare MBA, mCADRE and CORDA1 (hepatocyte model constructed using the same data as the mCADRE

reconstruction), here we also show these results for CORDA2, the reconstruction performed using the latest HPA data.

Amino-acid Recycling tests

We first tested the ability of the models to recycle 20 amino-acids and ammonia into urea. For this test, the models were allowed to uptake glucose, the basal inputs, and the amino-acid being tested. This then made the amino acid the only source of nitrogen in the model. The release rate of urea was then set to a strictly positive rate and optimized using Flux Balance Analysis. If the model returned a feasible flux distribution with a positive urea release rate, the test was considered passed. If no exchange reaction was present for the given amino-acid, the test was not performed and the result was deemed inconclusive. The results are presented in **STable3**:

	Recon1	MBA	CADRE	CORDA1	CORDA2
L-Glutamate	1	1	0	1	1
Glycine	1	1	0	1	1
L-Alanine	1	1	0	1	1
L-Lysine	1	1	0	1	1
L-Aspartate	1	1	1	1	1
L-Arginine	1	1	1	1	1
L-Glutamine	1	1	0	1	1
L-Serine	1	1	0	1	1
L-Methionine	1	1	-1	1	1
L-Tryptophan	1	1	-1	1	1
L-Phenylalanine	1	1	-1	1	1
L-Tyrosine	1	1	0	1	1
L-Cysteine	1	1	0	1	1
L-Leucine	1	1	1	0	1
L-Histidine	1	1	1	1	1
L-Proline	1	1	1	1	1
L-Asparagine	1	1	-1	-1	-1
L-Valine	1	1	-1	1	1
L-Threonine	1	1	1	1	0
L-Isoleucine	1	1	1	1	1
Ammonium	1	1	1	1	1

STable3: Amino-acid recycling test results. Red colored squares (-1) indicate a failed test and green colored squares (1) indicate a passed test. Blue colored squares (0) indicate the exchange reaction being tested was not present in the model.

Glucogenic Tests

Next we tested the ability of each of the models to produce glucose from 21 different metabolites. First we tested the ability of each model to produce glucose from the basal metabolites only. None of the models was able to do so. For the glucogenic tests, the model was then allowed to uptake the basal inputs and the metabolite being tested. The production of glucose was then optimized using Flux Balance Analysis. If the model was able to produce glucose from the source provided, the test was considered passed. If the exchange reaction for the given metabolite was not present in the model, the test was not performed and the result was deemed inconclusive. The glucogenic test results are present in **STable4**:

	Recon1	MBA	CADRE	CORDA1	CORDA2
L-Glutamate	1	1	0	1	1
Glycine	1	1	0	1	1
L-Alanine	1	1	0	1	1
L-Aspartate	1	1	1	1	1
L-Arginine	1	1	1	1	1
L-Glutamine	1	1	0	1	1
L-Serine	1	1	0	1	1
L-Methionine	-1	-1	-1	-1	-1
L-Tryptophan	1	1	-1	1	1
L-Phenylalanine	1	1	-1	1	1
L-Tyrosine	1	1	0	1	1
L-Cysteine	1	1	0	1	1
L-Histidine	1	1	1	1	1
L-Proline	1	1	1	1	1
L-Asparagine	1	1	-1	-1	-1
L-Valine	1	1	-1	1	1
L-Threonine	-1	-1	-1	-1	0
L-Isoleucine	1	1	1	1	1
L-Lactate	1	1	0	1	1
Glycerol	1	1	0	1	1
Pyruvate	1	1	-1	1	1

STable4: Glucogenic test results. Red colored squares (-1) indicate a failed test and green colored squares (1) indicate a passed test. Blue colored squares (0) indicate the exchange reaction being tested was not present in the model.

We find that two of the metabolic tests were not passed by any of the models or Recon1. Given the hepatocyte-specific models are subsets of Recon1, none of the models would be able to pass these tests no matter how comprehensive they were. For that reason, we consider only 19 of the 21 tests in the main text.

Nucleotide Production Tests

Finally, the ability of each model to produce 8 different nucleotides was tested. For these tests, the models were allowed to uptake the basal inputs, glucose, and ammonium (as a source of nitrogen). A sink consuming the nucleotide being tested was then added to the cytosolic compartment and optimized using Flux Balance Analysis. If a positive flux through the sink was allowed, the test was considered passed. Since no exchange reactions were tested here, none of the tests could be inconclusive. The test results are presented in **STable5**:

	Recon1	MBA	CADRE	CORDA1	CORDA2
ATP	1	-1	1	1	1
CTP	1	1	1	-1	1
dATP	1	-1	1	1	1
dCTP	1	1	1	1	1
dGTP	1	-1	1	1	1
dTTP	1	1	1	-1	-1
GTP	1	-1	1	1	1
UTP	1	1	1	1	1

STable5: Nucleotide production test results. Red colored squares (-1) indicate a failed test and green colored squares (1) indicate a passed test. These metabolic tests were included in the mCADRE reconstruction process.

Exchange Reactions

The MBA reconstruction contained 247 exchange reactions, while mCADRE contained 197, CORDA1 249, and CORDA2 226. **STable6** indicates whether the exchange reactions of some basic metabolites are present in either reconstruction. We can see that CORDA reconstructions contain considerably more basic exchange reactions.

	MBA	mCADRE	CORDA2	CORDA2
Hydrogen peroxide	1	1	1	1
Water	1	0	1	1
Protons	0	0	1	1
Ammonium	1	1	1	1
Nitric oxide	1	1	1	1
Oxygen (O_2)	0	0	1	1
Superoxide anion	1	1	1	1
Carbon Dioxide	1	0	1	1
Sulfate	1	0	1	1
Phosphate	1	1	1	1
ATP	1	1	1	1
GTP	1	1	1	1
GDP	0	0	1	1
AMP	0	0	1	1
GMP	0	0	1	1
NAD	1	0	0	0
NADP	1	0	0	0
Sodium	0	0	0	0
5,6,7,8-Tetrahydrofolate	1	1	1	1
Urea	1	1	1	1
Bicarbonate	1	0	1	1
Hexadecanoate	0	0	1	1
D-Lactate	1	1	1	1
L-Lactate	1	0	1	1
Linoleic acid	1	1	1	1
Octadecanoate	1	1	1	1
Triacylglycerol	1	1	1	0
Cholesterol	1	0	1	1

STable6: Exchange reaction of basic metabolites present in each model.

Calculation of Healthy and Cancer-specific Models

Recon 2 modifications

Before models were calculated, four reactions were modified in the Recon2 reconstruction. The reactions *r1402* and *r1403* were added back into the model to allow for glycogen primer production and degradation respectively, and the reactions *ARTPLM2 and ARTPLM3* were added back to allow for the production of lipids and glycerides. A primer glycogenin (*ggn*) exchange reaction was also added (Tyr_ggn[e] <=> Tyr_ggn[c]) to allow for the consumption and production of glycogen.

HPA data

To perform the tissue-specific reconstructions, proteomics data was downloaded from the Human Protein Atlas[1,2] for both healthy and cancer tissue. In both cases, only proteins with *supportive* reliability were used. Healthy tissue proteomics data was expressed in terms of Not detected, Low, Medium and High expression, and models were calculated in the same manner as described in the main text. Briefly, using the gene-reaction association rules, gene names were replaced with numerical values of -1, 0, 1, 2, and 3, corresponding to Not Detected, not in the dataset, Low, Medium and High expression. Next, OR gates were replaced with MAX, AND gates were replaced with MIN, and the expression was evaluated. Reactions with final scores of -1 were assigned to the NC group, 1 and 2 scores were assigned to the MC group, and scores of 3 were assigned to the HC group. When multiple protein expression levels were given for a single protein, the highest level was used. A total of 32 metabolic tests were also assigned to the HC group and tested one at a time (S1_Table).

Protein cancer data obtained from the HPA database was expressed differently. For each protein in each cancer type, expression levels were assigned for any number of samples analyzed. In order to assign a single protein score to each protein, numerical values were assigned to each sample as -1, 1, 2, and 3 corresponding to Not detected, Low, Medium and High respectively. The mean value between all calculated samples was then used in the gene-reactions association rules, and reaction scores were calculated the same manner as before. Reactions with a mean protein score less than or equal to -0.5 were assigned to the NC group, greater than 1 and less than 2.5 to the MC group, and greater than or equal to 2.5 to the HC group. Distribution of reactions groups can be observed in figure **SFig7**. While evidence for fewer proteins was used to calculate the cancer models, their distribution was similar.
SFig8 and **SFig9** show the number of reactions from each group used to calculate the model (left), and ultimately included in the model (right), for both healthy and cancer tissues respectively. We can see a general pattern of inclusion of nearly all MC reactions and very few NC reactions in all models.
SFig10 and **SFig11** demonstrate reaction inclusion distributions in healthy and cancer tissues respectively. We can see a larger core set of reactions, as well as a small number of reactions included only in a few models.

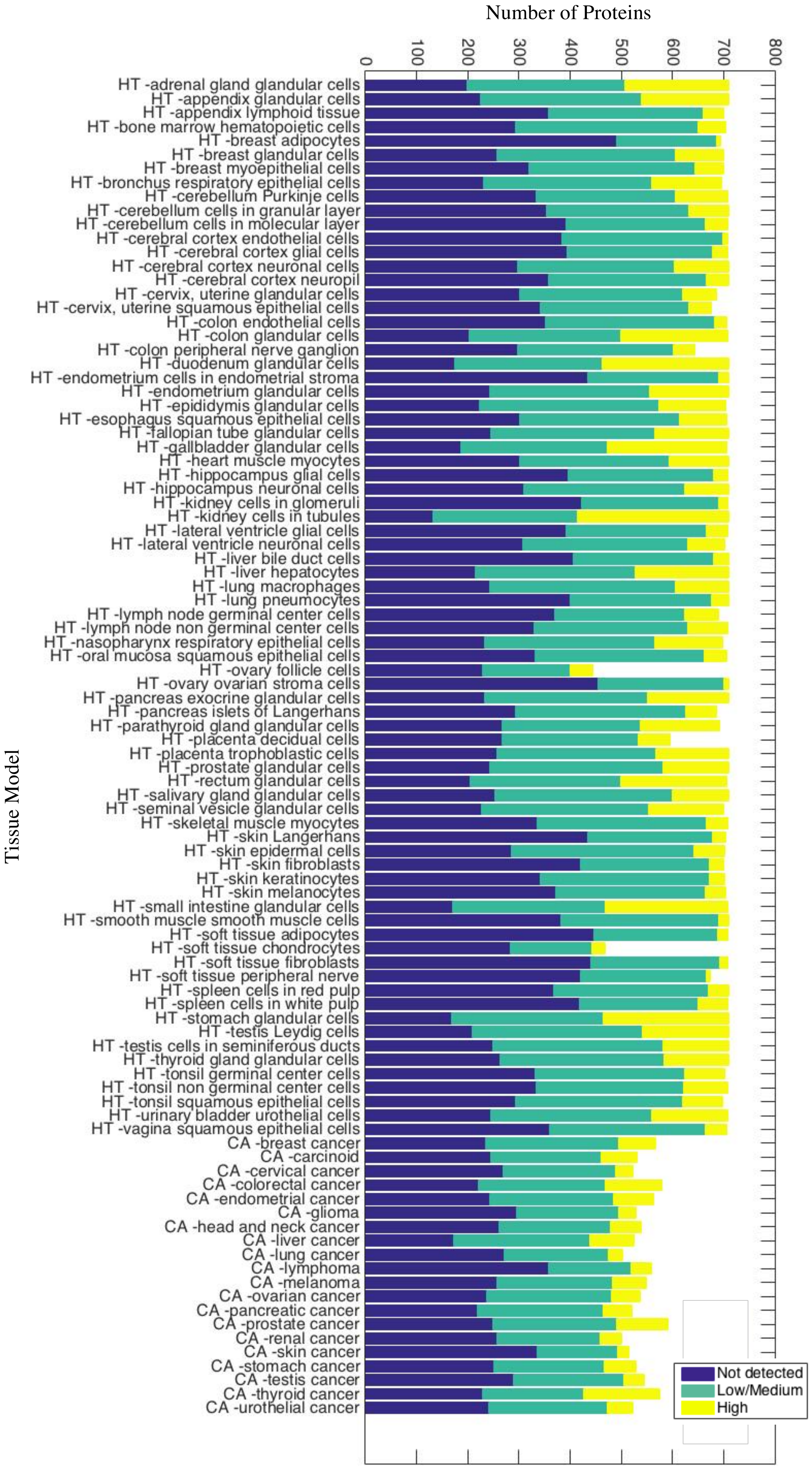

SFig7: Distribution of protein evidence scores used to calculate healthy and cancer tissue models. Abbreviations: HT – healthy tissue, CA – cancer.

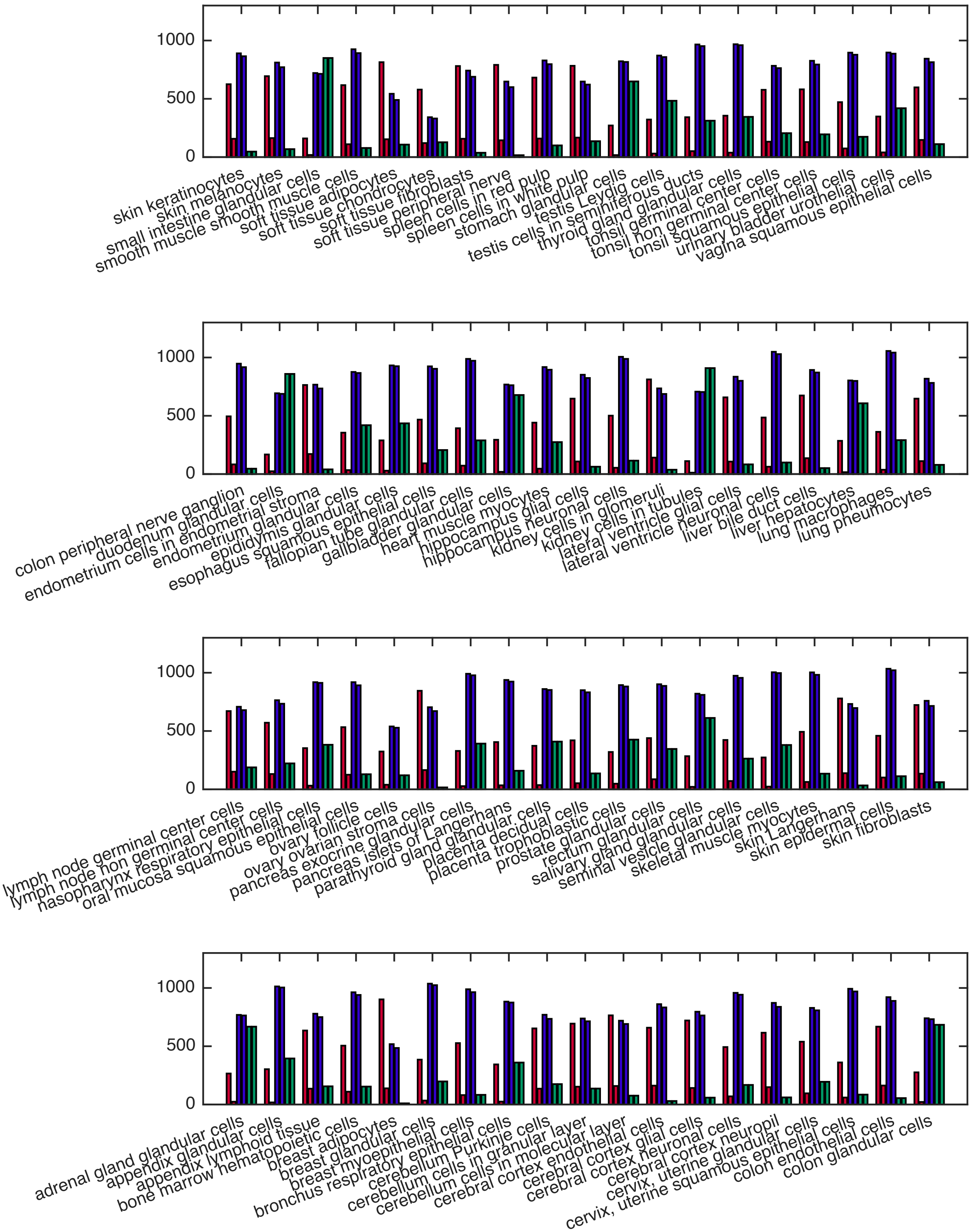

SFig8: Distribution of reaction category used to calculate each healthy tissue model. NC reactions are in red, MC reactions in blue and HC reactions in green. Left bars show all reactions used as input to the CORDA algorithm, and right bars show all reactions ultimately included in the model.

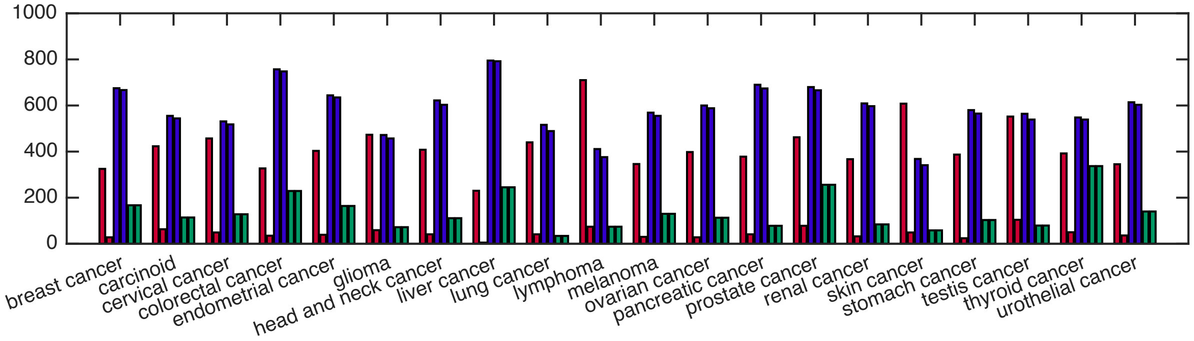

SFig9: Distribution of reaction category used to calculate each cancer tissue model. NC reactions are in red, MC reactions in blue and HC reactions in green. Left bars show all reactions used as input to the CORDA algorithm, and right bars show all reactions ultimately included in the model.

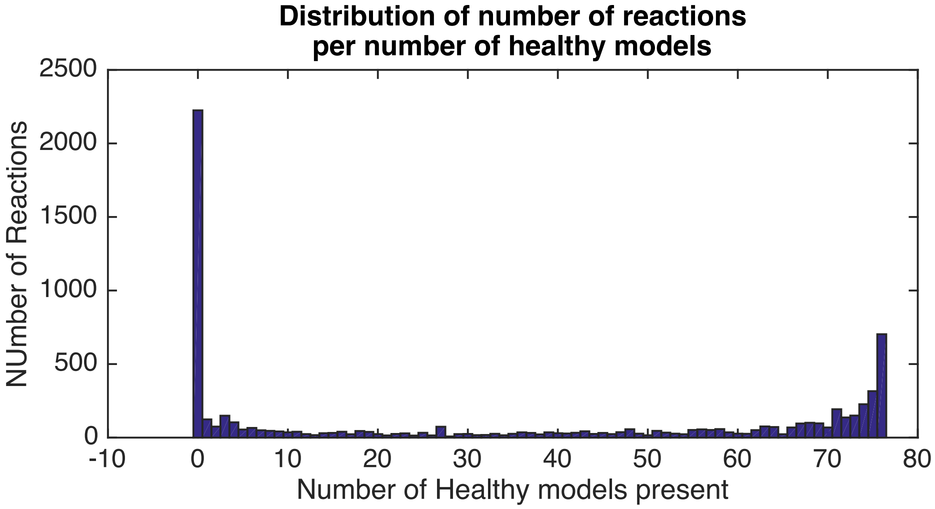

SFig10: Distribution of reactions included per number of model in healthy tissues. Y–axis indicates number of reactions in each group, and x-axis indicates number of models in which reactions in that group are present.

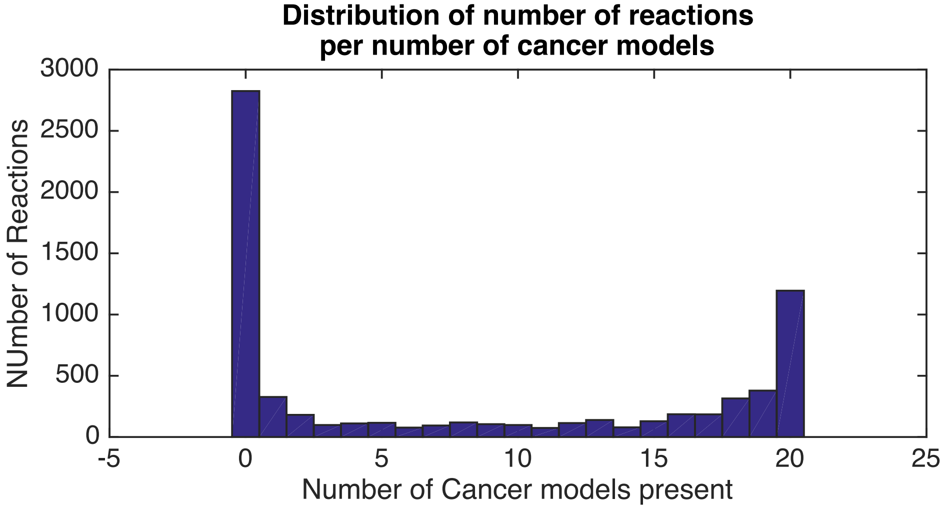

SFig11: Distribution of reactions included per number of model in cancer tissues. Y–axis indicates number of reactions in each group, and x-axis indicates number of models in which reactions in that group are present.

HMG-CoA Reductase Functionality

During Monte-Carlo sampling analysis of hepatocyte specific models, the reaction HMGCOARx was shown to be always active, and present at relatively high flux values in the MBA reconstruction. To investigate the effect of this reaction on each hepatocyte model, we determined which of the remaining model reactions are dependent on HMGCOARx. This was done by setting HMGCOARx's upper and lower bounds to zero and determining which reactions lost their ability to carry flux as a result. Overall, 188 reactions were dependent on HMGCOARx in the MBA liver model, 156 reactions in mCADRE, 30 in CORDA1 and 33 in CORDA2. The distribution of these reactions per subsystem is presented in **STable7**.

	MBA	mCADRE	CORDA1	CORDA2
**Cholesterol Metabolism**	25	28	22	25
**Transport, Peroxisomal**	44	22	4	4
**Bile Acid Biosynthesis**	31	37	0	0
**Exchange**	12	13	0	0
**Transport, Endoplasmic Reticular**	6	10	4	4
**Transport, Extracellular**	9	11	0	0
**Fatty acid oxidation, peroxisome**	17	0	0	0
**Glyoxylate and Dicarboxylate Metabolism**	9	3	0	0
**Carnitine shuttle**	6	3	0	0
**Transport, Mitochondrial**	2	7	0	0
**Fatty acid activation**	6	1	0	0
**Lysine Metabolism**	6	0	0	0
**Taurine and hypotaurine metabolism**	1	5	0	0
**Glycine, Serine, and Threonine Metabolism**	2	1	0	0
**Purine Catabolism**	3	0	0	0
**CoA Biosynthesis**	0	3	0	0
**Vitamin D**	0	3	0	0
**Alanine and Aspartate Metabolism**	1	1	0	0
**Arginine and Proline Metabolism**	1	1	0	0
**Fatty acid elongation**	2	0	0	0
**Fatty Acid Metabolism**	1	1	0	0
**Nucleotides**	2	0	0	0
**Pentose and Glucuronate Interconversions**	2	0	0	0
**CoA Catabolism**	0	2	0	0
**R Group Synthesis**	0	2	0	0
**Miscellaneous**	0	1	0	0
**Sphingolipid Metabolism**	0	1	0	0
**Total:**	188	156	30	33

STable7: Number of reactions, per subsystem, which are blocked due to the removal of HMGCOARx in each model.

The blockage of HMG-CoA reductase should block the ability of the models to produce *de novo* cholesterol. Therefore, the high number of reactions in cholesterol metabolism that are blocked is expected. The blockage of reactions in other unrelated subsystems, however, such as amino-acid and nucleotide metabolism, is unexpected. This analysis further supports the idea that HMGCOARx plays a more fundamental role in the parsimonious models, and demonstrates how this minimalistic approach can lead to the elimination of alternative pathways.

Monte-Carlo Sampling

Monte-Carlo sampling was performed in each model in the tissue-specific library, and healthy and cancer tissue models were presented together in the main text. **SFig12** shows the boxplots for each model in the library in the reactions considered in the main text, as well as GHMT2r(m). This plot demonstrates the high variability in each of these pathways between the different tissue models.

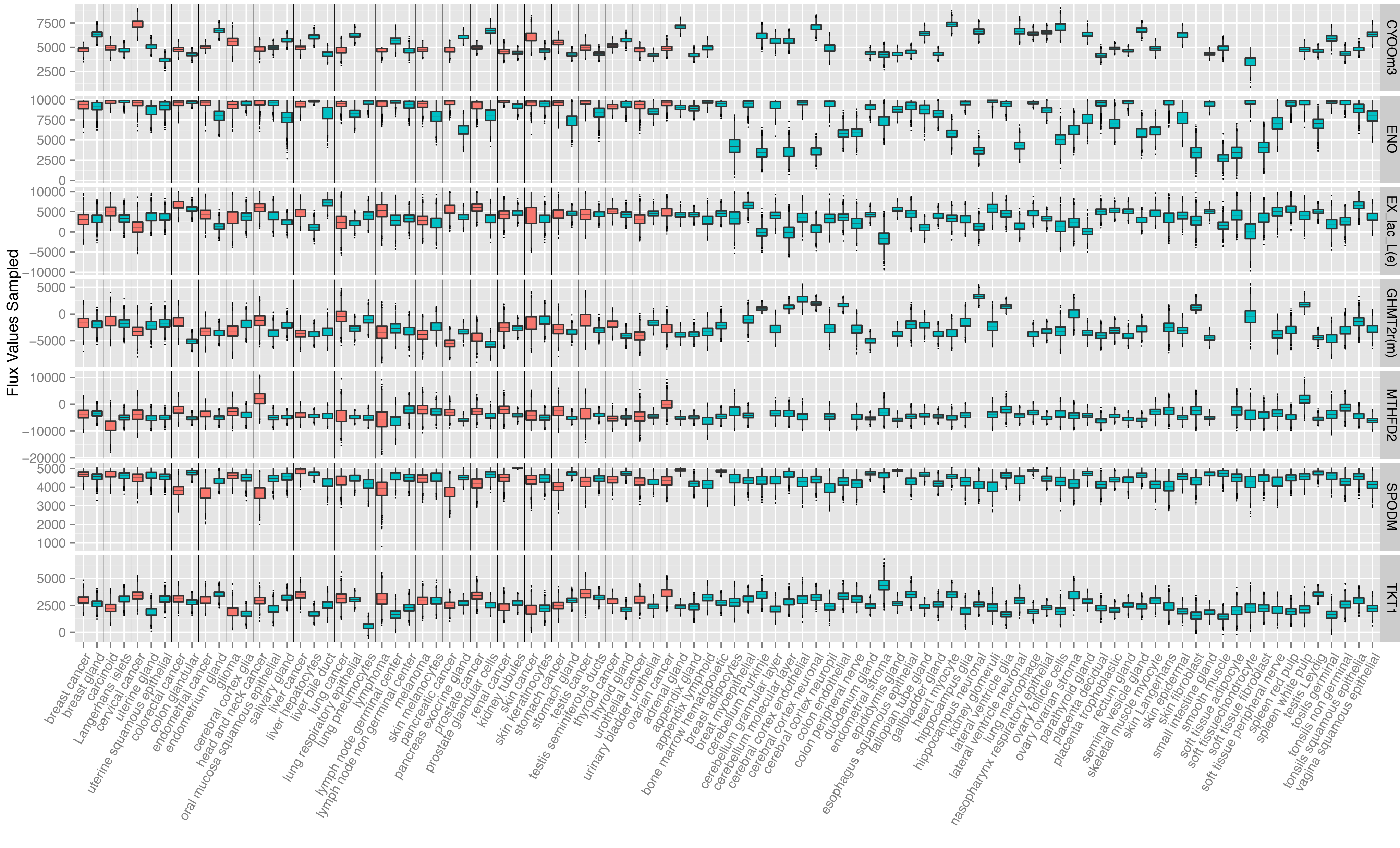

SFig12: Boxplot of sampled flux values for individual models of reactions considered in the main text and GHMT2r. Red boxes indicate cancer models and green boxes indicate healthy tissue models. Horizontal lines group cancer models with their healthy counterparts (top). Models below the last horizontal line do not have a related cancer or healthy counterpart.

Here we also analyze the activity of GHMT2r and GHMT2rm in cancer and healthy tissue models, and compare this activity with the expression of its associated gene *SHMT2*. While *SHMT1* is also associated with these reactions, the expression evidence for *SHMT1* is classified as *uncertain* in the HPA, while *SHMT2* is supportive. For this reason, *SHMT2* was used in the model reconstructions and *SHMT1* was not. When looking at the activity of these reactions in cancer versus healthy tissue models, we find a very similar overall distribution (**SFig13**).

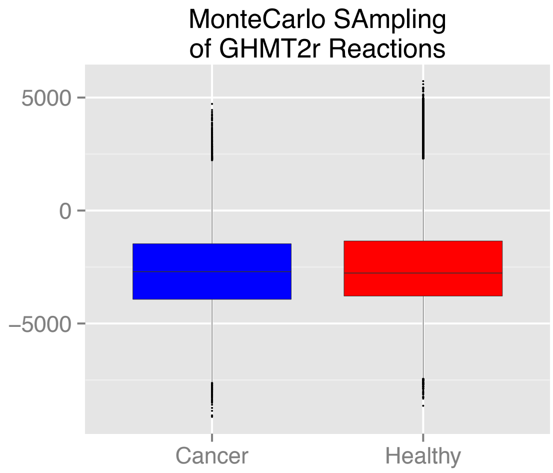

SFig13: Flux distribution through GHMT2r and GHMT2rm in cancer versus healthy tissue models shows a similar distribution.

As mentioned in the main text, while the activity of these reactions has been shown to be generally up-regulated in cancer tissues, this differential expression has been shown to be heavily dependent on cell type. Considering this fact, here we evaluate each cancer model separately, and compare the activity through the *SHMT2* related reactions to their healthy counterparts. Based on protein expression of *SHMT2* in cancer models, we have identified seven cancer types that have a significantly higher expression of this protein in cancer versus their healthy tissue counterparts. To identify these tissues, we calculated the protein score for *SHMT2* in each of the cancer models as described in the main text. These scores were then divided by the *SHMT2* score in the cancer tissue's healthy counterpart. *SHMT* was considered up regulated for scores of 1.17 or higher. Results from this calculation are available in **STable8**.

Cancer Type	Protein Evidence in the Human Protein Atlas				Cancer Score	Healthy Tissue Score	Cancer/ Healthy
	High	Medium	Low	Not Detected			
Breast	8	4	0	0	2.67	2	1.33
Carcinoid	1	2	0	1	1.50	2	0.75
Cervical	5	6	0	1	2.17	2	1.08
Colorectal	9	0	0	0	3.00	3	1.00
Endometrial	5	2	2	3	1.50	2	0.75
Glioma	1	8	2	1	1.67	1	1.67
Head and Neck	1	2	0	0	2.33	2	1.17
Liver	1	6	2	3	1.17	3	0.39
Lung	5	4	1	1	2.09	1.5	1.39
Lymphoma	10	2	0	0	2.83	3	0.94
Melanoma	3	7	1	0	2.18	2	1.09
Pancreatic	1	8	2	0	1.91	2	0.95
Prostate	1	6	1	1	1.67	2	0.83
Renal	1	7	1	3	1.25	2	0.63
Skin	3	6	2	0	2.09	2	1.05
Stomach	6	5	0	0	2.55	2	1.27
Testis	7	4	0	0	2.64	1	2.64
Thyroid	3	0	1	0	2.50	2	1.25
Urothelial	1	8	0	2	1.55	2	0.77

STable8: Identification of *SHMT2* overexpression in cancer over healthy tissue model counterparts.

We then evaluated the sampled flux values through GHMT2r and GHMT2rm for cancers with over expression of *SHMT2* compared to other cancer types (**SFig14**). We can see that aside from the glioma model, all other models identified as over expressing *SHMT2* present sampled flux distributions shifted towards higher values, converting serine to glycine as opposed to glycine to serine. The conversion of serine to glycine has been shown to be a major source of carbon for the one-carbon metabolism required for the synthesis of nucleotides in cancer, occupying a key intersection between serine and glycine metabolism and nucleotide biosynthesis[3]. Furthermore, it is worth noting that glioma is the only cancer model where the *SHMT2* expression shifted from low to medium levels, which could explain this different result.

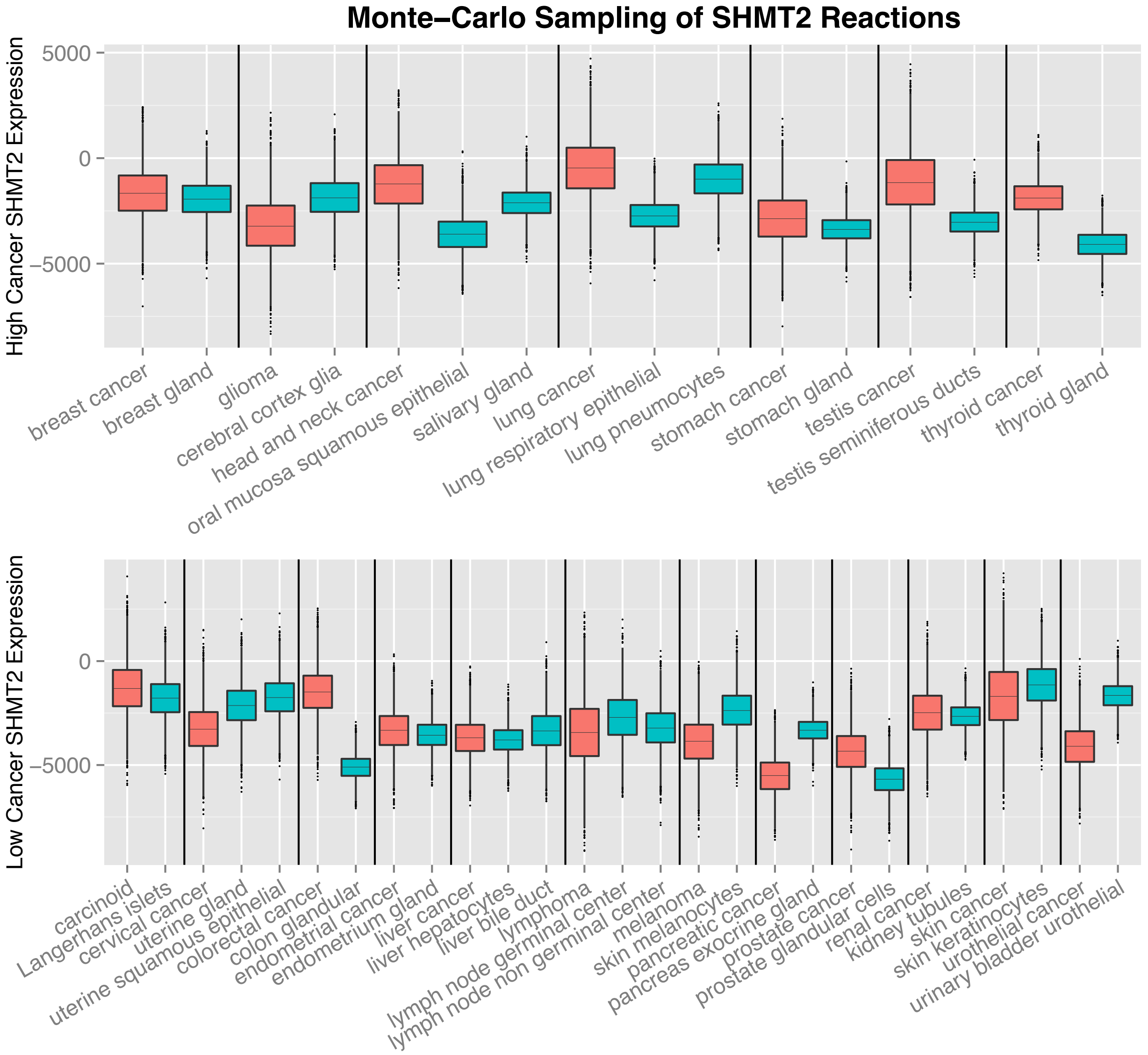

SFig14: Flux values sampled through GHMT2r and GHMT2rm for cancers with high (top) and medium-low (bottom) expression of *SHMT2* according to the HPA. In general, cancers with high expression of *SHMT2* showed a higher capacity of conversion of serine to glycine.

Overall, this analysis indicates the ability of the CORDA algorithm to identify functionality in specific types of cancer models when compared to their healthy counterparts, not only of cancer compared to healthy tissue models in general.

Citations

1. Uhlen M, Oksvold P, Fagerberg L, Lundberg E, Jonasson K, Forsberg M, et al. Towards a knowledge-based Human Protein Atlas. *Nat Biotechnol*. 2010 Dec;28(12):1248–50.
2. Uhlen M, Fagerberg L, Hallstr¨om BM, Lindskog C, Oksvold P, Mardinoglu A, et al. Proteomics. Tissue-based map of the human proteome. *Science*. 2015 Jan;347(6220):1260419.
3. Amelio, I., Cutruzzola, F., Antonov, A., Agostini, M., & Melino, G. (2014). Serine and glycine metabolism in cancer. *Trends in biochemical sciences*, *39*(4), 191-198.
