# Supplementary material for: Reconstruction of Tissue-Specific Metabolic Networks Using CORDA
Source: PLoS Comput Biol. 2016 Mar 4;12(3):e1004808. doi: 10.1371/journal.pcbi.1004808 (PMC4778931; doi:10.1371/journal.pcbi.1004808)
Supplement: S2 Text — Example of application of CORDA to small sample networks. (PDF) [file pcbi.1004808.s002.pdf]

Here we will exemplify steps 1 and 2 of the CORDA tissue building algorithm and some of its features using small sample networks. Parameters used during this example are: ***Γ** = 10,000, **κ** = 1e-02, **ε** = 1, **n*** = 5, and ***p** = 2.* Throughout these examples we will use the underline notation 1,000, for example, to denote a particular number plus the defined noise (a number sampled at random between 0 and ***κ***).

Step1:

To exemplify the first step in the tissue building algorithm, consider the network defined in **S2Fig1**:

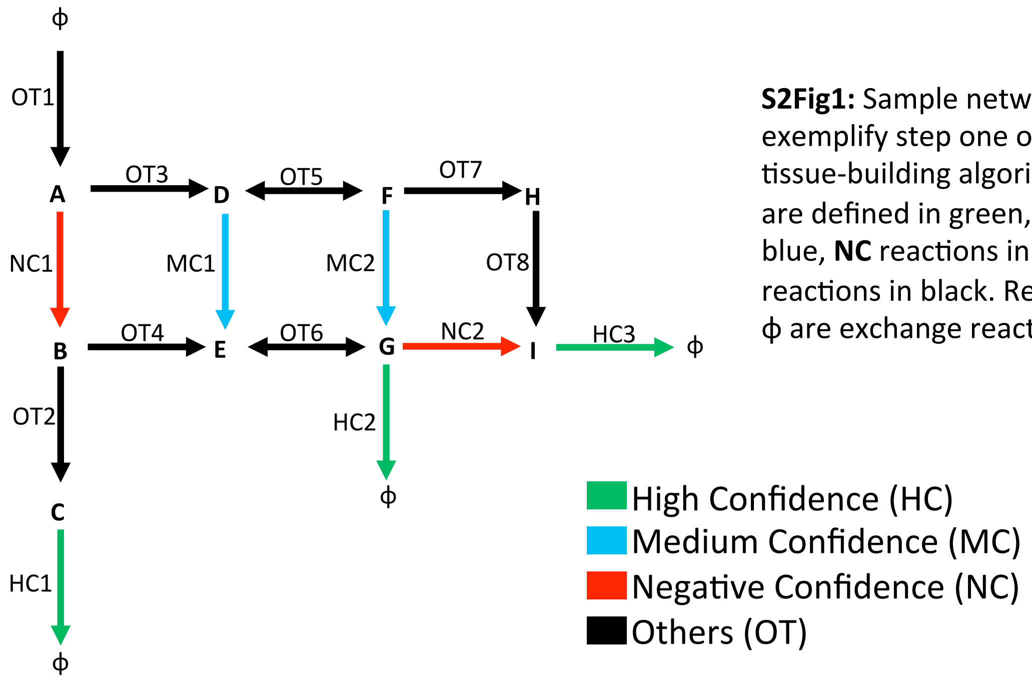

S2Fig1: Sample network used to exemplify step one of the CORDA tissue-building algorithm. **HC** reactions are defined in green, **MC** reactions in blue, **NC** reactions in red, and **OT** reactions in black. Reactions involving ϕ are exchange reactions

During each dependency assessment, the network above is modified in the following manner (**S2Fig2**):

**Reactions:**	1	**Reactions:**	2	**Reactions:**
**OT1:** ϕ -> A		**OT1:** ϕ -> A + *0*e		**OT1:** ϕ -> A + *0*e
**OT2:** B -> C		**OT2:** B -> C + *0*e		**OT2:** B -> C + *0*e
**OT3:** A -> D		**OT3:** A -> D + *0*e		**OT3:** A -> D + *0*e
**OT4:** B <-> E		**OT4:** B -> E + *0*e		**OT4:** B -> E + *0*e
**OT5:** D <-> F		**OT5f:** D -> F + *0*e		**OT5f:** D -> F + *0*e
		**OT5b:** F -> D + *0*e		**OT5b:** F -> D + *0*e
**OT6:** E -> G		**OT6f:** E -> G + *0*e		**OT6f:** E -> G + *0*e
		**OT6b:** G -> E + *0*e		**OT6b:** G -> E + *0*e
**OT7:** F -> H		**OT7:** F -> H + *0*e		**OT7:** F -> H + *0*e
**NC1:** A -> B		**NC1:** A -> B + *10,000*e		**NC1:** A -> B + *10,000*e
**NC2:** G -> I		**NC2:** G -> I + *10,000*e		**NC2:** G -> I + *10,000*e
**MC1:** D -> E		**MC1:** D -> E + *100*e		**MC1:** D -> E + *100*e
**MC2:** F -> G		**MC2:** F -> G + *100*e		**MC2:** F -> G + *100*e
**HC1:** C -> ϕ		**HC1:** C -> ϕ + *0*e		**HC1:** C -> ϕ + *0*e
**HC2:** G -> ϕ		**HC2:** G -> ϕ + *0*e		**HC2:** G -> ϕ + *0*e
**HC3:** I -> ϕ		**HC3:** I -> ϕ + *0*e		**HC3:** I -> ϕ + *0*e
				**CONS:** e -> ϕ

S2Fig2: Reaction modifications performed during the first step of the CORDA algorithm.

1. First, reversible reactions (such as **OT5** and **OT6** in this example) are split into forward and backwards reactions. Next, a new pseudo-metabolite, denoted here by *e*, is added as being produced by each reaction. The stoichiometric coefficient of *e* defines the *"cost"* associated with each reaction. Each **NC** reaction receives a cost of Γ and each **MC** reaction receives a cost of $\sqrt{\Gamma}$. Next, the cost of each reaction is increased by a random number sampled uniformely between zero and κ. In the end, **NC** reactions will receive a cost in the order of 1e+04, **MC** reactions in the order of 1e+02, and the remaining reactions in the order of 1e-02.

2. Next, an extra reaction is added to the model, denoted in the previous figure by **CONS**. This reactions consumes the cost metabolite *e*. This way, while every reaction in the model produces this cost, this added reactions is the only one that consumes it. This reaction is also set as the model objective.

The stoichiometric matrix *S* of the model changes in the following manner (**S2Fig3**):

	HC1	HC2	HC3	MC1	MC2	NC1	NC2	OT1	OT2	OT3	OT4	OT5	OT6	OT7	OT8
A						-1		1		-1					
B						1			-1		-1				
C	-1								1						
D				-1						1		-1			
E				1							1		-1		
F					-1							1		-1	
G		-1			1		-1						1		
H														1	-1
I			-1				1								1

	HC1	HC2	HC3	MC1	MC2	NC1	NC2	OT1	OT2	OT3	OT4	OT5	OT6	OT7	OT8	CONS
A						-1		1		-1						
B						1			-1		-1					
C	-1								1							
D				-1						1		-1				
E				1							1		-1			
F					-1							1		-1		
G		-1			1		-1						1			
H														1	-1	
I			-1				1								1	
e	*0*	*0*	*0*	*100*	*100*	*10,000*	*10,000*	*0*	*0*	*0*	*0*	*0*	*0*	*0*	*0*	-1

S2Fig3: Transformation of stoichiometric matrix defined in figure 1 used to calculate reaction dependency assessments.

After tailoring the model, each reaction in **HC** is evaluated. That is, the upper and lower bounds of that reaction are set to $\pm\boldsymbol{\varepsilon}$ (which is one in this example), and FBA is performed while minimizing the flux through **CONS**. Here we evaluate the results for each **HC** reaction in the sample network:

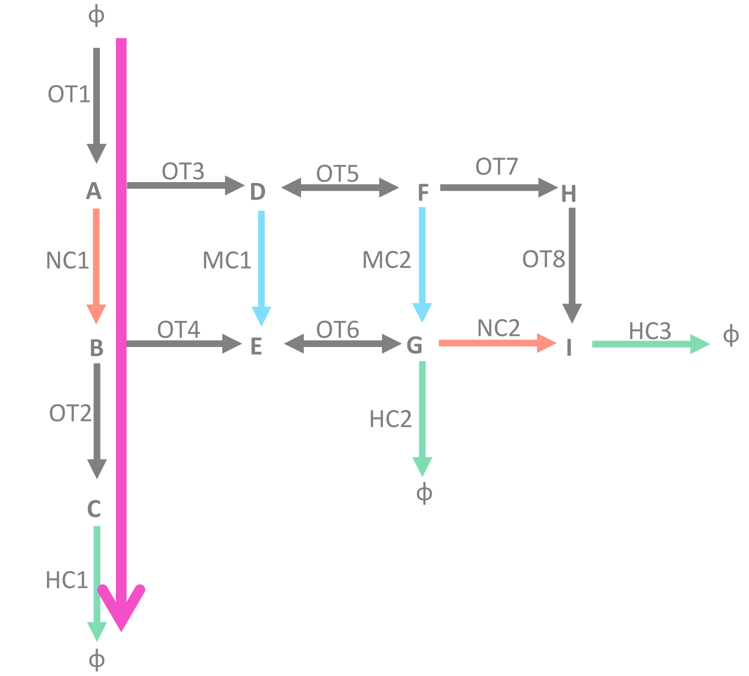

S2Fig4.1: The first high confidence reaction **HC1** is dependent on the negative confidence reaction **NC1**. That is, if **NC1** were to be removed from the network, **HC1** would not be able to carry flux. Therefore, the pathway highlighted in pink must be taken, and the reaction **NC1** would be moved to the tissue model.

S2Fig4.2: For the second high confidence reaction, two different pathways have an overall cost in the order of 1e+02. These pathways are highlighted in pink in the diagram below. The pathway taken in this case will depend on the noise added during the reaction dependency assessment. By performing the assessment multiple times (n > 1), pathways with the same cost are samples. The pathway highlighted in blue, however, will not be taken, since it has a cost in the order of 1e+04. In this case, since n=5, both **MC1** and **MC2** will most likely be moved to the tissue model.

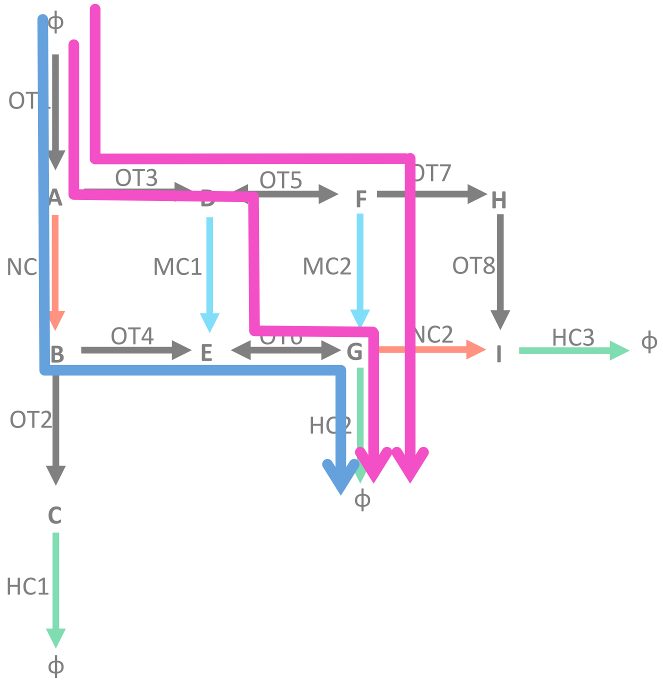

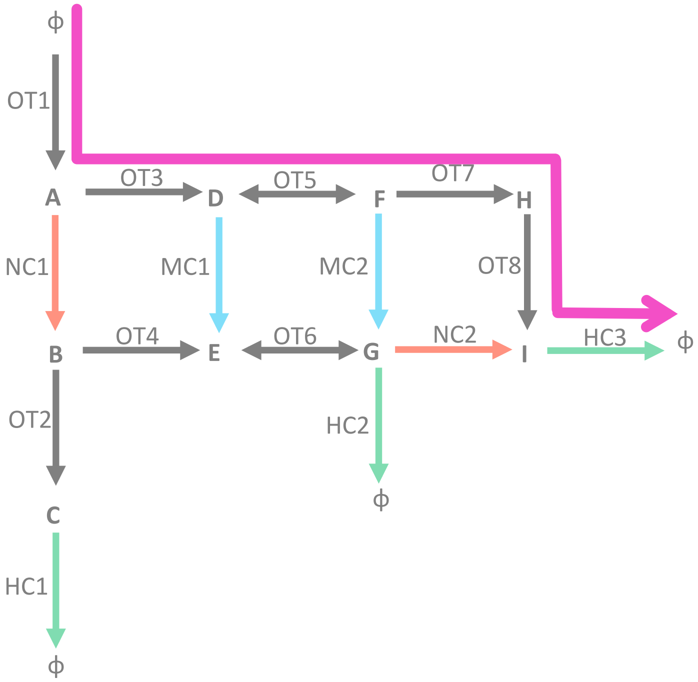

S2Fig4.3: The third high confidence reaction does not depend on any **MC** or **NC** reactions. This reaction will then not lead to the inclusion of any additional reactions to the tissue model at this point in the algorithm. The pathway highlighted on the top has a cost in the order of *1e-02*, while alternative pathways would have a higher cost.

In this first step, reactions **MC1**, **MC2** and **NC1** would be added to the **RE** group. The reaction **NC2** was not associated with any **HC** reaction, and will therefore not be included in the final model (at least not at this point. Next, we exemplify step two of the algorithm.

Step 2:

To exemplify step 2 of the tissue-building algorithm, consider the network depicted in figure **S2Fig5**. At this point, **NC** reactions receive a cost of *Γ*, and all other reactions receive only the added noise. We will not exemplify the network transformation since it follows a logic similar to the transformation depicted in **step 1**.

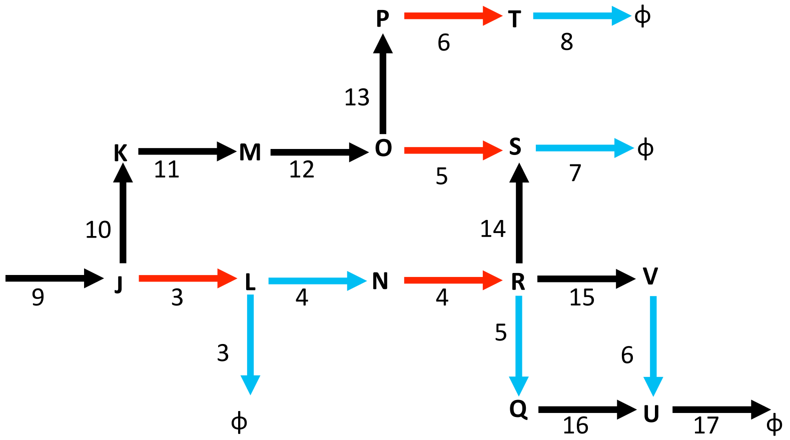

S2Fig5: Sample network used to exemplify step two of the CORDA tissue-building algorithm. **MC** reactions are defined in blue, **NC** reactions in red, and **OT** reactions in black. Reactions involving ϕ are exchange reactions

In the next two steps, we identify the association between MC and NC reactions:

S2Fig6.1:
As defined by the highlighted pathways on the right, **MC3** is associated with **NC3** only, while **MC5** and **MC6** are associated with both **NC3** and **NC4**.

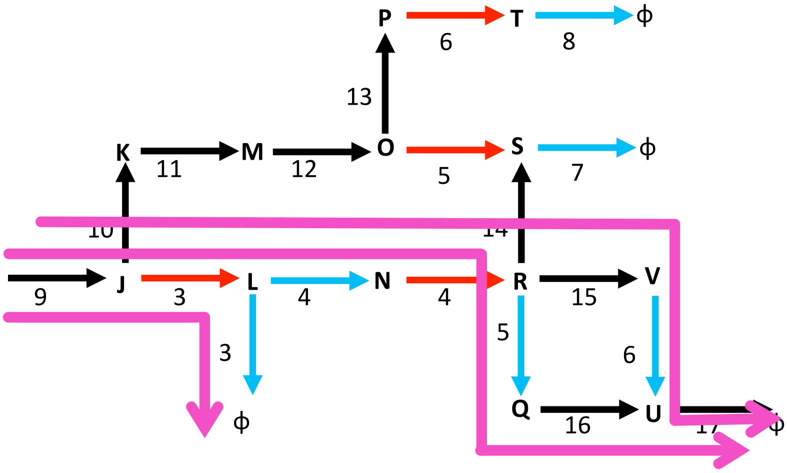

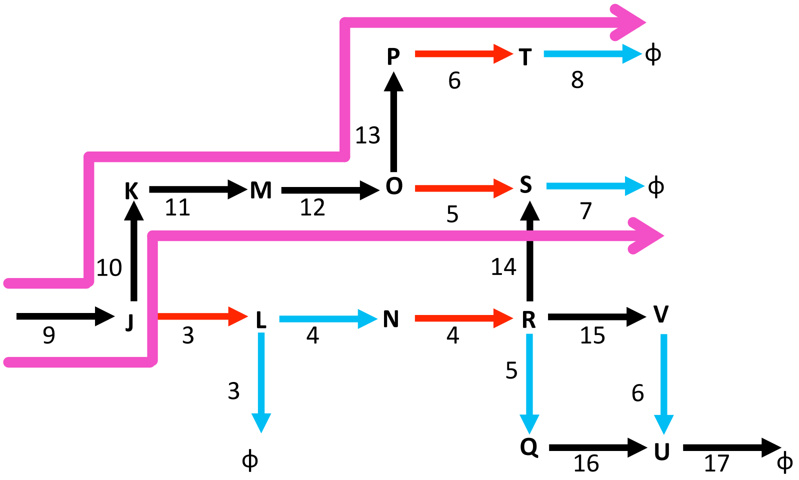

S2Fig6.2:
MC7 and **MC8** are associated with negative confidence reactions **NC5** and **NC6** respectively. It is worth noting that reaction **MC7** can carry flux through **NC3**, **MC4**, **NC4** and **OT14**. This pathway, however, would have a cost in the order of 2e+4, while the pathway highlighted on the left has a cost in the order of 1e+4.

Based on these reaction associations, we can define the association matrix between **MC** and **NC** reactions defined below:

	MC3	MC4	MC5	MC6	MC7	MC8
NC3	1	1	1	1		
NC4		1	1	1		
NC5					1	
NC6						1

Since in this example, we set *p=2*, reactions **NC3** and **NC4** are moved into the **RE** group, while reactions **NC5** and **NC6** are blocked in the metabolic network. Next, we see which **MC** reactions are still able to carry flux. Since **NC3** and **NC4** are still both present in the model, the pathways present in **S2Fig6.1** are still present, and reactions **MC3**, **MC4**, and **MC6** are moved to the **RE** group. For reaction **MC7**, although it's associated **NP** reaction was blocked, this reaction is still able to carry flux, but with the use of two **NP** reactions as opposed to one (**S2Fig7**). Reaction **MC7** is then moved to the **RE** group. Reaction **MC8**, however, is blocked through the blockage of **NP6**, and is not realocated to the **RE** group.

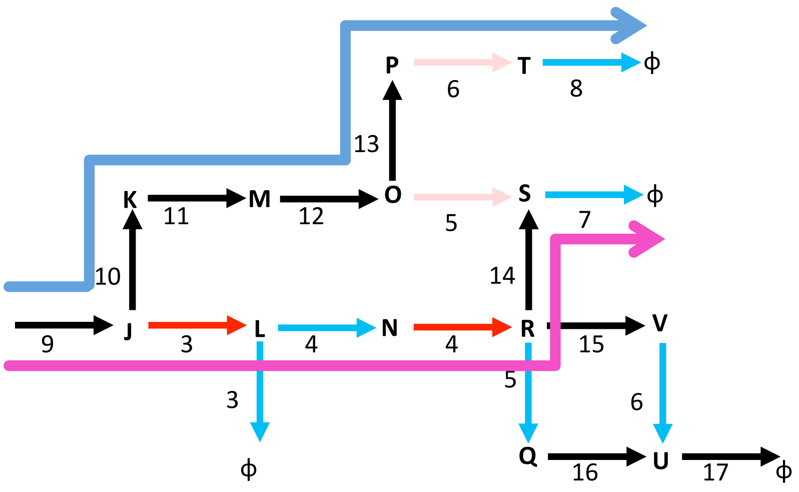

S2Fig7: Sample pathways for reactions **MC7** and **MC8** following the blockage of **NP** reactions associated with less than *p* **MC** reactions. The pathway highlighted in pink is available, moving **MC7** to the **RE** group, while the pathway highlighted in blue is no longer available, removing **MC8** from the final reconstruction.

There are two main reasons we have chosen to take this approach in step two over simply including **NP** reactions associated with *p* or more **MC** reactions, and their corresponding **MC** reactions. First, this was done in order to maximize the number of **MC** reactions included in the tissue reconstruction. As mentioned in the main text, and exemplified here, if we had taken the alternative approach, reaction **MC7** would not be present in the final tissue model, since it's associated **NP** reaction is associted with fewer than *p* **MC** reactions. This reaction, however, is still able to carry flux following the blockage of the remainig **NC** reactions, and can be moved to the **RE** group in order to maximize the inclusion of **ME** reactions. Secondly, an **MC** reaction can be associated with muliple **NP** reactions, some of which are associated with *p* or more **MC** reactions and some of which are not. In the alternative approach, there is no clear answer as to whether to include these **MC** reactions or not, while with the approach taken here this problem is avoided.
